# Demand driven salt clean-up in a molten salt fast reactor – Defining a priority list

**DOI:** 10.1371/journal.pone.0192020

**Published:** 2018-03-01

**Authors:** B. Merk, D. Litskevich, R. Gregg, A. R. Mount

**Affiliations:** 1 University of Liverpool, School of Engineering, Liverpool, United Kingdom; 2 National Nuclear Laboratory, Chadwick House, Warrington, United Kingdom; 3 The University of Edinburgh, EaStCHEM, School of Chemistry, Edinburgh, United Kingdom; Los Alamos National Laboratory, UNITED STATES

## Abstract

The PUREX technology based on aqueous processes is currently the leading reprocessing technology in nuclear energy systems. It seems to be the most developed and established process for light water reactor fuel and the use of solid fuel. However, demand driven development of the nuclear system opens the way to liquid fuelled reactors, and disruptive technology development through the application of an integrated fuel cycle with a direct link to reactor operation. The possibilities of this new concept for innovative reprocessing technology development are analysed, the boundary conditions are discussed, and the economic as well as the neutron physical optimization parameters of the process are elucidated. Reactor physical knowledge of the influence of different elements on the neutron economy of the reactor is required. Using an innovative study approach, an element priority list for the salt clean-up is developed, which indicates that separation of Neodymium and Caesium is desirable, as they contribute almost 50% to the loss of criticality. Separating Zirconium and Samarium in addition from the fuel salt would remove nearly 80% of the loss of criticality due to fission products. The theoretical study is followed by a qualitative discussion of the different, demand driven optimization strategies which could satisfy the conflicting interests of sustainable reactor operation, efficient chemical processing for the salt clean-up, and the related economic as well as chemical engineering consequences. A new, innovative approach of balancing the throughput through salt processing based on a low number of separation process steps is developed. Next steps for the development of an economically viable salt clean-up process are identified.

## Introduction

When considering demand driven development of energy technologies, the current big challenge of energy research is the energy ‘trilemma’. The trilemma is described (e. g. by the world energy council [1], an UN-accredited global energy body) as addressing the challenges to reduce emissions, to enhance security of supply, and to reduce the cost of energy production. This description coincides very well with UN Goal 7: “Ensure access to affordable, reliable, sustainable and modern energy for all as one piece of sustainable development of the future world “[2]. An additional request can be put onto the agenda for nuclear energy; this is to solve the nuclear waste problem which has been created by the operation of earlier reactor generations in electricity production. Nuclear fission has the potential to become a major low carbon energy source but based on this, clear cost reduction would be one of the crucial requirements.

Innovative nuclear reactors, like those promoted by the Generation IV International Forum [3], operated in the so-called ‘closed fuel cycle’ can provide a promising solution for two of these challenges of the trilemma since nuclear reactors have the potential to assure sustainable energy security and efficiency by providing reliable infrastructure based on highly innovative systems. The application of these closed fuel cycle operated systems offers an attractive solution to acute threat of climate change. However, a disruptive step in the development of nuclear reactors will be required to capitalize the benefits more efficiently and in a more economical way than with established reactor technologies. One proposal is the development of a molten salt fast reactor operating on spent fuel from existing nuclear power plants to enforce a circular economy in nuclear [4] and solve the long term activity and storage problems in the nuclear waste [5]. Besides the development of the fast reactor system, reprocessing and production of the nuclear fuel are key points to address in establishing the currently proposed closed fuel cycle [6]. Molten salt systems offer here a clear potential for cost reduction due to the possibilities for optimization of the re-processing/salt clean-up as well as due to the elimination of solid fuel production. This has been identified to be the major cost driver in the closed fuel cycle for sodium cooled fast reactor technology [7].

As it is stated in the book «Reprocessing and Recycling of Spent Nuclear Fuel» edited by Robin Taylor: ‘The roots of nuclear reprocessing also are found in that realm of the war and commercial technology. After Seaborg and coworkers discovered plutonium, it was quickly established as an alternative pathway to nuclear weapons that could be built around nuclear reactors and chemical reprocessing’ [8]. Thus, the key demand was to get hands on the plutonium produced in a reactor, via the chain reaction, using chemical processing. Following initial processes (the BiPO_4_ process and an initial REDOX process based on nitric acid media), the first promising process was the PUREX (Plutonium Uranium Redox EXtraction) process. PUREX was developed at the end of the 1940s and is still the backbone of civil, industrial reprocessing technologies [8]. Several facilities for the reprocessing of commercial nuclear fuel have been built since this time in Russia, UK, France, Japan, and India, all based on the PUREX technology. Nowadays, a new challenge has arisen after development of the waste management technology partitioning and transmutation (P&T). The key chemical step, the partitioning of the materials is partly still based on the PUREX process, but with additional downstream process steps like the DIAMEX and one of the SANEX processes [9, 10]. To avoid possible proliferation concerns which arise from the pure separated plutonium fraction, new processes like COEX or GANEX are under development for future application [9]. Another process to be mentioned is TALSPEAK which has been proposed for the selective extraction of the trivalent lanthanides from the actinides in aqueous solution [11].

All these processes have a common demand, they require incredibly high recovery factors, ideally 99.9% or better to avoid the carryover of fissile materials and transuranium (TRU) isotopes, especially into the waste stream for final disposal. Any possible carryover would poison the gain of the P&T process due to the accumulation of TRUs in the waste stream caused by the required multiple recycling.

The disruptive innovation of molten salt fast reactors, ideally operating directly on spent nuclear fuel from LWRs, has the potential to become a game changer for P&T [12, 13] as well as for closed fuel cycle operation with reprocessing for electricity production [4, 5]. The integrated technology would allow more demand driven solutions since no time demanding and costly intermediate steps are required [4, 5]. Thus, the invention of a highly innovative reactor system also creates the opportunity for a disruptive innovation in reprocessing technology. In this special case the phrase reprocessing won’t be the right description anymore, as ‘fuel chemistry is an area of vital importance to a fluid-fuel reactor comparable to fuel structure, cladding integrity, and coolant stability in a solid-fuel reactor.’[14] This requires a salt cleaning or chemical processing system. The historic salt cleaning system developed as part of MSRE fuel chemistry was based on fluorination to recover Uranium [14]. Thus, it was developed to fulfil the same principles as accessing fissile materials. However, as already mentioned, a more demand driven solution could be possible.

One key step for a disruptive process development in salt clean-up is the identification of the main chemical elements which prevent the reactor from long term operation due to a strong negative influence on either the neutron balance in the reactor or the chemical behavior of the fuel. The physical issues are considered by addressing the question: ‘Which elements produced during the fission reaction have, due to their amount and their properties, the strongest negative influence on reactor criticality?’ In the second part of the article, we will discuss the optimization opportunities which are given by an integrated system with a direct link between reactor and salt clean-up.

The work will be opened by a discussion of the possibilities given by this innovative technology. There follows a numerical study of the influence of different chemical elements in the molten salt fast reactor, based on a light water reactor spent fuel configuration. Both parts will be drawn together to create a priority list followed by a discussion of the opportunities and challenges as basis for a demand driven, interdisciplinary research and development of the salt clean-up system.

## Possibilities for innovative technology

The molten salt fast reactor technology opens up possibilities for innovation due to the integrated technology, with co-location of the reactor and the fuel cycle facility and the opportunity to rethink reprocessing based on the demand for a chemical separation technology to allow long term reactor operation in a closed cycle [4, 5]. This arrangement even allows us to leave the beaten track of separating the fissile materials. A demand driven view on the problem dictates the challenge as ‘Take out what prevents the reactor from long term sustainable operation’ instead of recovering the fissile material to produce new fuel. This approach has big potential since it can solve several historic problems including:

- Avoiding/reducing proliferation risk,
- Avoiding losses of TRU into the waste stream
- Avoiding specific requirements for the partitioning of minor actinides like in the P&T strategies
- Avoiding the manual handling of highly radioactive TRUs in solid fuel production, while allowing all TRUs to stay in the system.

Some important boundary conditions of molten salt reactor operation create new opportunities which lead to new options for the separation technology. A fraction of the molten salt containing the fission products will be split continuously from the reactor and has to be cleaned in some way. This is in strong contrast to today’s batch working approach of unloading the fuel, storing it in the cooling pond, transporting it to the reprocessing facility into another storage pond, and finally dissolving the solid fuel in concentrated nitric acid [8] to produce the raffinate containing fissile materials which can be used to produce new solid fuel. After the separation of a fuel stream in the molten salt reactor, the cleaned stream is directly re-fed into the reactor in a continuous process. There is no transfer of the separated fissile materials to the fuel fabrication plant, no solid fuel fabrication, no transport to the nuclear power plant, and no fresh fuel storage. The fission products separated from the salt stream offer in addition a great opportunity for novel, specific and optimized conditioning for long term storage if this is required due to their long term activity.

However, the integrated approach offers not only new opportunities but also new challenges. Due to the elimination of the storage times, the process must be designed as a ‘hot process’, working under a high irradiation level. The process chemistry should be based on the molten salt which is used as fuel solvent in the reactor since a transformation would be too complex, resource intensive and expensive. Thus, the temperature of the operation of the salt clean-up should be on a level comparable to the inlet temperature of the reactor to avoid segregation of the dissolved chemical elements or even a freezing of the salt. Another important point is the criticality safety of the fuel salt during reprocessing. The salt split from the reactor must be treated in a criticality safe process which requires a high neutron leakage, since there isn’t the possibility of diluting the solution like in classical aqueous reprocessing. In addition, the process has to be very reliable since it has to be operated almost continuously and at constant efficiency to avoid a request for extensive storing of salt on the one hand, or (through a breakdown of the clean-up system) undermining reliable reactor operation through fission product poisoning on the other hand. Thus the salt clean-up is a key factor, since the cleaned salt stream will act as continuous feed for the reactor, which will not possess excess reactivity, unlike classical nuclear reactors. Besides these challenges, there are promising opportunities. No special fuel preparation following reprocessing would be needed if the separation process works on the molten salt. The amount of the salt split from the reactor can be freely chosen, thus the material flow can be adapted and optimized to achieve the separation rate desired in the chemical processes. No separation of fissile materials is required since there is no demand for solid fuel production. In the ideal process, the fissile material should even be untouched since this reduces the proliferation concerns as well as the carryover of fissile materials into the waste stream. No handover of fissile material with the risk of proliferation as well as theft and misuse should or will happen anymore. These opportunities offer the chance to design a completely new, demand driven, more economic separation system based on significantly lower separation factors and thus reduced process demands, and with new optimization potential. However, a deeper understanding of the influence of the fission product elements on the reactor neutron economy is required to develop a disruptive process; the first step is to be able to develop a priority list of elements to be separated from the molten salt fuel. Reflecting the influence of the different elements on the system criticality of a molten salt reactor core, the impact of the different chemical elements is created by a combination of the amount of the element in the fuel salt and its absorption cross section. Knowledge of this combination will be produced in the following numerical study.

## Data, methods, and models

In order to evaluate the influence of different chemical elements on the nuclear molten salt reactor’s neutronics a slightly modified model of the molten salt fast reactor (MSFR) proposed in the EVOL [15] benchmark was chosen as the ‘workhorse’ system. This configuration has already been considered in several previous publications, e. g. [4, 5, 12, 13].

### Geometry of the reactor

**Fig 1:**
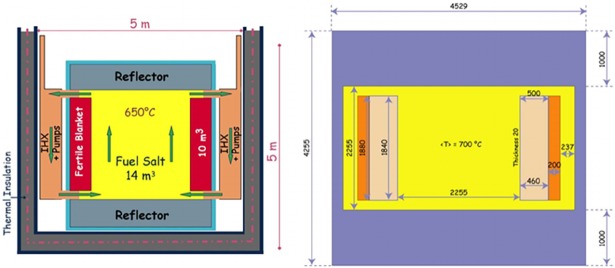
Geometry of the model reactor used in this study.

The reference MSFR core proposed in the EVOL project is a 3000 MW_th_ reactor with a fast neutron spectrum. The publicly available illustration of the benchmark geometry of the molten salt fast reactor is shown in Fig 1.

As in the EVOL model, the core is a single cylinder where the nuclear reactions occur within the flowing fuel salt. The core is composed of three volumes: the active core, the upper plenum, and the lower plenum. In contrast to the EVOL benchmark, the fertile salt component was excluded from this study as the fertile blanket was only filled with an inactive surrogate (pure LiF salt).The radial reflector has a volume of 7.7 m^3^ and is surrounded by a neutronic protection consisting of B_4_C, see Fig 1. The temperature of the fertile salt, reflector and structural materials in this study was chosen to be 1000 K.

### Initial salt composition

The fuel salt considered in the simulations is a salt consisting of lithium fluoride, fission products and plutonium as major fissile material. The composition of the salt is given as LiF—(FPF3 –PuF_3_, whose FP:Pu proportion was set at 35 mole %. The choice of this fuel salt composition was based on the data from the EVOL benchmark and relies on many systematic studies which have been necessary to achieve the self-sustained operation in SNF on the longer term [4, 5]. This salt composition leads to a fast neutron spectrum in the core. The operating temperature was set to 1000 K, which is close to the average 973 K proposed in the EVOL benchmark. The density of the salt was recalculated in accordance with the formula given in the EVOL benchmark as equal to 4.085 g/cm^3^. The same value was used for the salt in the blanket region.

The composition of the heavy nuclei used in this study consisted of fission products from LWR burnt fuel and Pu as fissile material. The following fission products were included into the composition of the fuel salt: Z = 30, 31, 32, 33, 34, 35, 37, 38, 39, 40, 48, 49, 50, 53, 55, 56, 57, 58, 59, 60, 61, 62, 63, 64, 65, 66, 67, 68, 69, 70. The basic composition of the different investigated spent fuel (including molar fraction and mass of each isotope) has been calculated with the code FISPIN [19]. The following spent fuel compositions have been supplied by the National Nuclear Laboratory for this study:

- freshly extracted from the reactor LWR spent fuel with burnup 30 GWd/tHM
- LWR spent fuel after 10 years in the pool (burnup 30 GWd/tHM)
- freshly extracted from the reactor LWR spent fuel with burnup 50 GWd/tHM
- LWR spent fuel after 10 years in the pool (burnup 50 GWd/tHM)
- freshly extracted from the reactor AGR spent fuel with burnup 25 GWd/tHM
- AGR spent fuel after 10 years in the pool (burnup 25 GWd/tHM)

For this publication we have decided the most probable future SNF configuration to be that in bold (LWR, 50 GWd/tHM) which is, due to its highest fission product concentration, also the most conservative choice (composition, see S1 File). The isotopic composition of the Pu vector used in this study (Table 1) is taken from [16] and is based on calculations of an average composition representative of the UK Pu stockpile in the year 2025.

**Table 1:** Plutonium vector used in calculations.

Nuclide	Content (wo%)
^238^Pu	0.25
^239^Pu	68.77
^240^Pu	26.70
^241^Pu	1.76
^242^Pu	2.52

It should be noted that ^241^Am, which is presented in work [16] was excluded from consideration in the plutonium vector, however there is a significant amount of ^241^Am in the spent fuel.

The material composition of the structural materials used in the present study is as defined in the EVOL benchmark. These data is presented in the S2 File.

### Computational tools

Computations using the MSFR model described above were carried out using OpenMC code [17]. OpenMC is a relatively new Monte-Carlo code developed by MIT with a focus on high-performance algorithms and modern software development practices. OpenMC can use continuous-energy cross sections as well as group wise cross-sections. It uses constructive solid geometry representation enabling high-fidelity modeling of nuclear reactors and other systems. OpenMC is available as free software under an open source license. Despite the fact that the code is relatively young, it is already being used in a number of advanced R&D projects including the Consortium for Advanced Simulation of LWRs and the ANL Center for Exascale Simulation of Advanced Reactors [18].

The isotopic composition of the LWR spent fuel has been evaluated using FISPIN, a fuel inventory code developed and maintained by the National Nuclear Laboratory [19]. For these calculations, few-group microscopic cross sections were calculated using an existing CASMO-4 lattice calculation for either a typical PWR or AGR UO_2_ assembly. As such, the relevant cross sections for select U and Pu nuclides are self-shielded. For nuclear reactions not modelled by CASMO-4 (e.g. those with negligible neutronic effect), the 69 group flux spectrum calculated by CASMO-4 for the fuel is used to condense infinitely diluted cross sections to the few-group energy structure required by FISPIN.

Once the cross section data has been prepared, FISPIN is used to perform a single representative inventory calculation for each case. For the PWR cases, the initial fuel is NIU (non-irradiated uranium) fuel, enriched to 3.0 w/o and 4.5 w/o for the 30 GWd/tU and 50 GWd/tU cases respectively. For completeness, the U-234 concentration has been determined using the PASREF code which attempts to model the precise inventory expected from a gas centrifuge cascade. Furthermore, typical impurities for LWR UO2 fuel as well as the oxide carrier have also been included in the initial fuel inventory. A constant 38 W/gU power density was assumed, recognizing the precise value assumed has little impact on the final inventory once those nuclides in secular equilibrium have decayed away.

JEFF-3.2 nuclear data library was used for calculations. The library, containing more than 400 isotopes at different temperatures, is freely available for download at NEA Databank web page.

The molten salt configuration follows the scoping studies done in [4, 5] with a given SNF share and the addition of Pu. The Pu content in the initial core is chosen to achieve a critical configuration. The methodology of the criticality search as well as critical concentration of the Pu are presented in the following chapter.

## Reactor core simulation results and discussion

The calculations were started by a criticality search. The criticality search was carried out by varying the concentration of the Pu content (with constant Pu vector) in the molten salt while the SNF content had been kept on the constant level which was required for the self-sustained long term operation [4]. The binary search algorithm is used for definition of the critical concentration of plutonium. The OpenMC method *search_for_keff* with *bisect* option was used for this purpose.

Different variants with different initial composition of the fuel salt and different statistics were considered in the present study. The parameters of these variants are summarized in Table 2.

**Table 2:** Summary of the variants used in the sensitivity study.

Variant number	Type of the model	Number of histories per batch	Critical Pu concentration, % mole	k_eff_	Standard deviation Δ_keff_
1	Extraction approach	20 000	0.05895	1.00038	0.00054
2	Extraction approach	1 000 000	0.05895	0.99996	0.00022
3	Addition approach	20 000	0.05724	1.00158	0.00045
4	Addition approach	100 000	0.05724	1.00091	0.00020
5	Addition approach	1 000 000	0.05716	1.00009	0.00005

The term “extraction approach” in Table 2 indicates the model with the full list of the fission products while “addition approach” relates to the “clean” composition (lithium fluoride, depleted uranium and plutonium only). More detailed information about the above mentioned models and the reasons to create will be given in the discussion. The number of the inactive and active batches was equal to 50 and 100 correspondingly for all variants. The given standard deviation of the calculation result is for the references cases, thus for the system including all fission products in the extraction approach and for the ‘clean’ reactor configuration.

**Fig 2:**
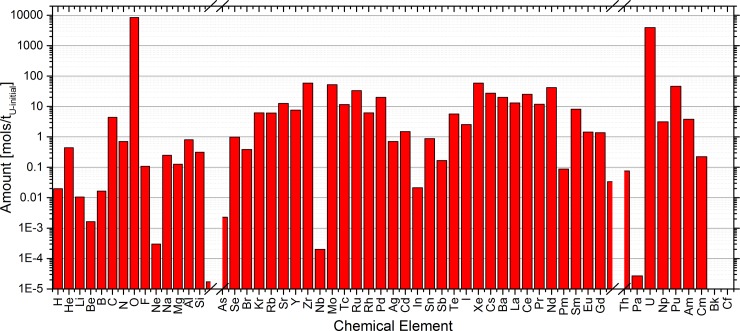
Elementary composition of the used spent nuclear fuel composition as calculated by FISPIN.

The spent LWR fuel with burnup equal to 50 GWd/tHM after 10 years in the pool contains more than 90 chemical elements. Each chemical element contains from 1 (F, Al) to 16 (Sn) isotopes. The SNF composition as given by a FISPIN [19] calculation is given in Fig 2. By far the majority of the SNF is still UO_2_, which typically is about 93 to 95% of the amount depending on the original enrichment and the burnup. However, the major fission product elements by amount can be identified in addition, they are Zr, Mo, Xe, and Nd, while the major breeding product is Pu. The full table with the SNF composition based on UOX fuel with a burnup of 50 GWd/tHM and a storage time of 10 years as given by FISPIN is presented in the supplementary materials.

**Fig 3:**
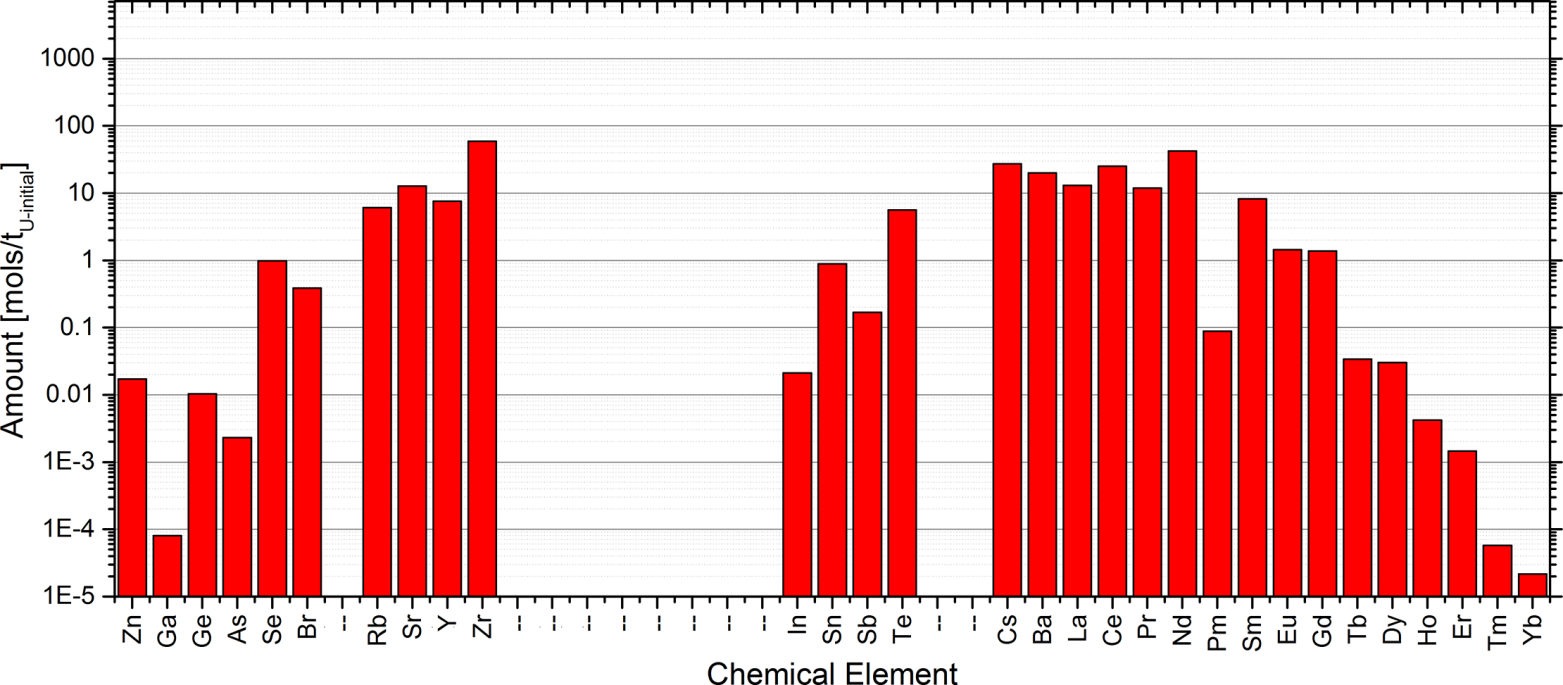
Fission products elements which are dissolved in the salt and are observed for the salt clean-up based on the list given in the EVOL benchmark.

Linking the information on the SNF with the list of elements considered in the EVOL benchmark for the salt clean-up provides the starting point for the following sensitivity study, which was performed based on the obtained critical composition. The elements and their amounts in the SNF are given in Fig 3. The dominant elements of the ones chosen to be used for the sensitivity study were Zr, Nd, Cs, Ce, and Ba.

**Fig 4:**
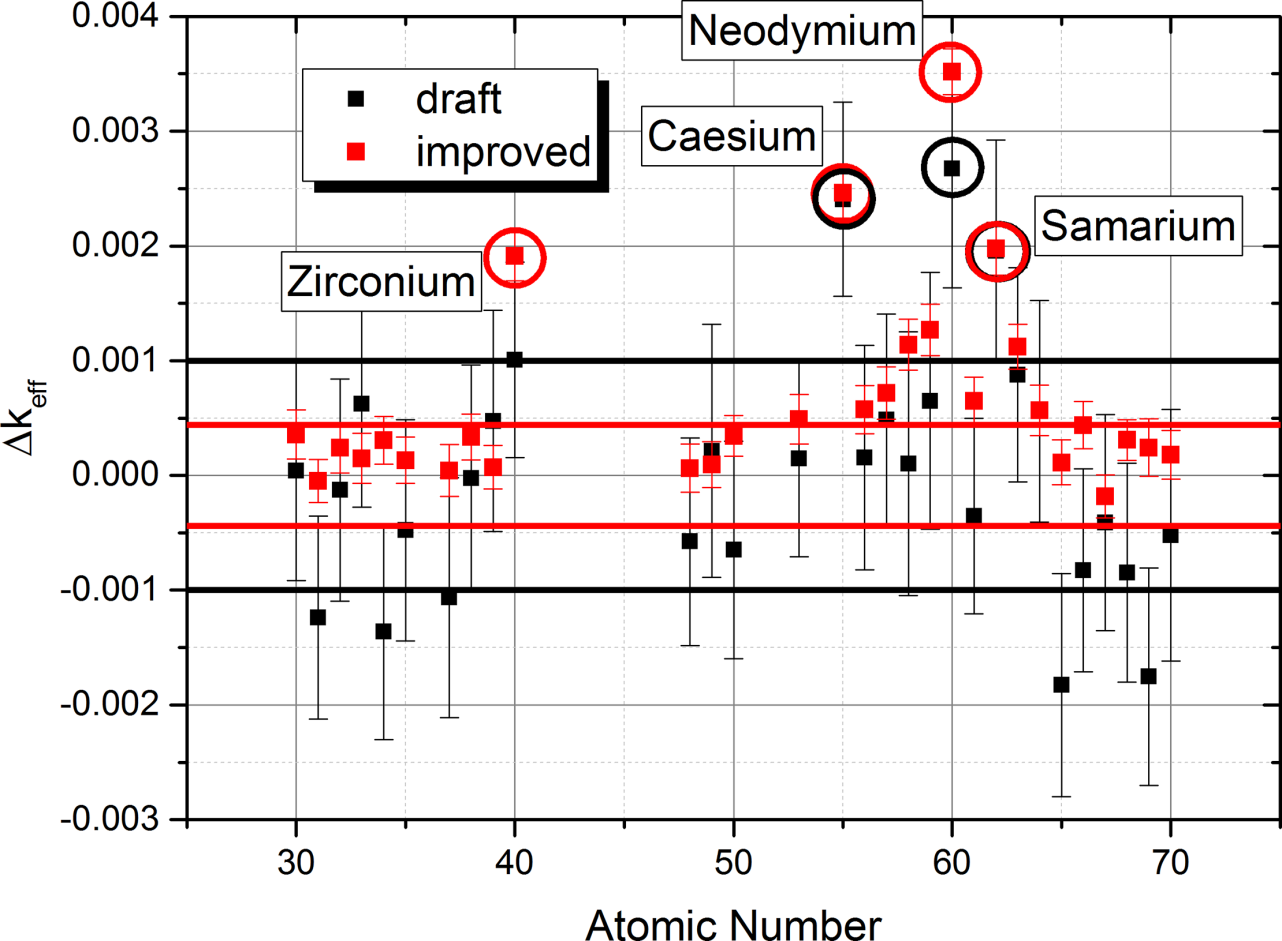
Sensitivity list for the observed chemical elements based on the EVOL benchmark element list using the extraction method for the simulation.

**Fig 5:**
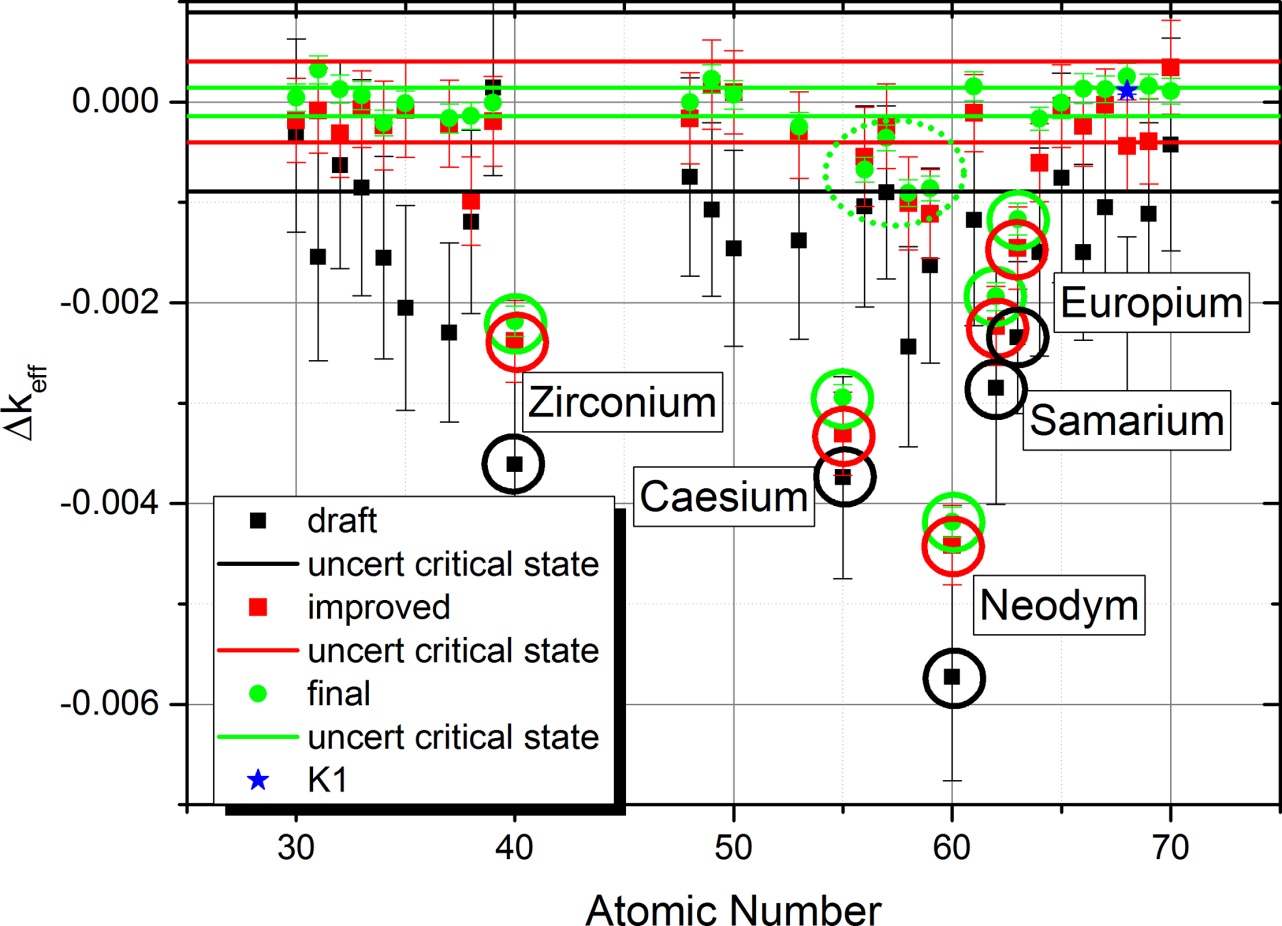
Sensitivity list for the observed chemical elements based on the EVOL benchmark element list using the addition approach for the simulation.

**Fig 6:**
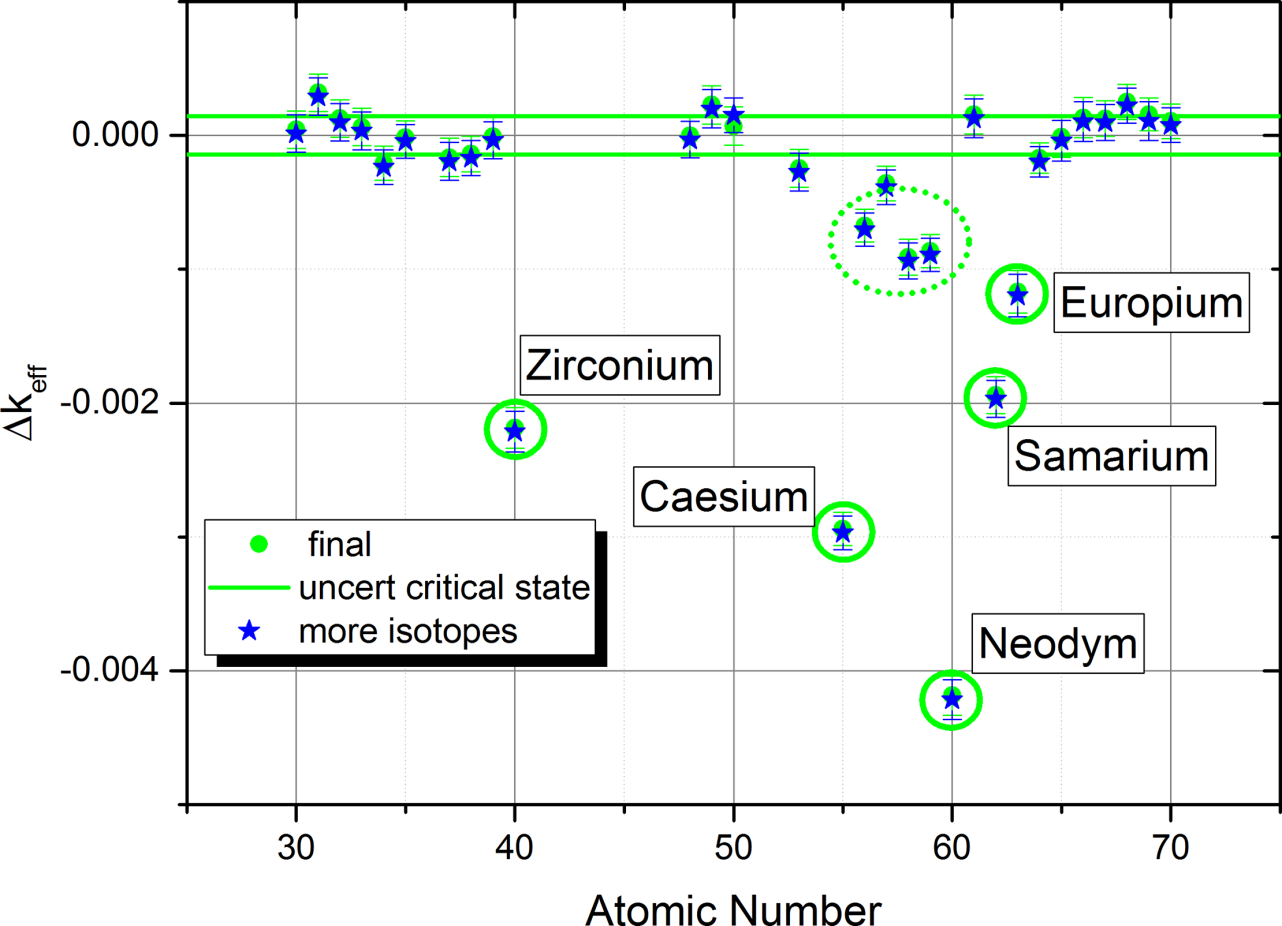
Cross check of the influence of the molar fraction cut-off on the sensitivity list.

When considering the list of isotopes given in supplementary materials, it is obvious that it will be almost impossible to run a Monte-Carlo calculation with acceptable computational demand and the requested accuracy. This led us to an important approximation, the cut-off of isotopes with low molar factions (below threshold of 10^−6^ [mols/t_U-initial_]). The standard deviations given in Table 2 have been used to determine the 95% confidence interval which is depicted by the black and red horizontal lines shown Fig 4 as well as the black, red and green lines in Fig 5 and Fig 6.

The initial “extraction approach” for the determination of the sensitivity list is based on the simulation of the extraction of each of the observed chemical element by reducing the content to 10% (extraction method). Only isotopes with a molar fraction higher than 10^−6^ are considered to achieve an acceptable computational performance. After this reduction the result for each calculation is compared to the reference case with all elements in the system. Based on this approach, an element with a high influence on the system criticality will lead to a positive Δk_eff_ for the system when extracted, since the neutron absorption is reduced. The results of a draft calculation using 20 000 histories per batch (black) and an improved setting with 100 000 particles per batch (red) are given in Fig 4 with the error bars for 95% confidence into the calculation result. Effective multiplication factors and standard deviations for these settings are presented in Table 2.

The applied approach give a first indication of the effect of the different chemical elements. However, a closer look indicates some problems with the simulation. The comparison of the results of the draft and the improved calculation for some of the elements e. g. 31, 34, 65, 66, 68, 69 show that there is no overlap of the error band. However, the error bands highlight the 95% confidence interval (i.e. plus/minus two standard deviations) of the calculated results, thus the number of outliers should be much lower. In addition, there are strong changes for important elements like, Zr and Nd. To improve the confidence, another refinement using higher statistics would be required, but the computational demand of this approach seems to be prohibitive.

This insight led us to a computationally much more efficient approximation, the “additive approach”, which allows us to improve the Monte-Carlo simulation and get a significant reduction of the standard deviation.

- draft: 20 000 histories per batch
- improved: 100 000 histories per batch
- final: 1 000 000 histories per batch

This approach is based on adding each element to a clean configuration consisting of the carrier salt, depleted uranium and the required Pu amount to achieve a critical configuration. This approach leads to a negative influence on Δ_keff_ for the system for elements which have a strong negative influence on the neutron economy. The final results (Fig 5) in green indicate now a really small uncertainty of the critical state and a clear indication not only of the clear contributors Zr, Cs, Nd, and Sm, but also the elements Eu, Ba, La, Ce, and Pr, which can be identified as having a clear contribution with no overlap of the error band with the uncertainty of the critical state. Thus the additive approach has given a more detailed insight into the problem. The only case where all error bands didn’t show an overlap (Erbium) has been investigated once more with improved settings (10 000 000 histories per batch, blue star) to achieve an overlap of the error bands.

The core approximation of all calculations is the cut-off of isotopes with a low molar fraction. To investigate the influence of this approximation a final test has been performed, reducing the cut-off level by a factor of 10 (i.e. isotopes with molar fraction higher than 10^−7^ were involved into calculations), see Fig 6. The results of the improved calculation using more isotopes (blue stars) do not show any significant change. Thus, the influence of the approximation seems to be limited for the investigated elements.

Evaluating the influence of the elements gives the following insight:

- Extracting the two major contributors Cs and Nd eliminates already almost 50% of the effect on criticality
- Extracting the 4 main contributors Cs, Nd, Zr, and Sm eliminates 78% of the effect on criticality
- Extracting in addition to the 4 contributors above Eu, Ce, and Pr eliminates 98% of the effect on criticality

From the separation chemistry point of view, Cs and Zr will be the elements which should be the likely focus of extraction. Nd belongs as lanthanide to the same group as U, and Sm belongs to the same group as Pu, thus their chemical properties are similar. This will form a significant challenge to the development of advanced separation methods which is foreseen to be a part of a future research programme on MSFR development. However, the following discussion of optimization highlights that the specific arrangement of molten salt reactors should confer advantages, including that the separation methods are not intrinsically required to achieve as high separation factors as classical reprocessing schemes. The elemental approach indicates that group extraction of lanthanides is unlikely to be a promising approach. In addition, this approach seems to be linked to a high risk of a carryover of actinides.

In general, in all applied cases the computational demand tends to infinity, thus an absolutely reliable result can’t be achieved with reasonable computational effort. There are two reasons for this: the very low content for some isotopes and the request for a significantly large number of reactions required in each isotope to reduce the error band to an acceptable dimension. This leads on the one hand to an increasing number of isotopes with the desired improvement of accuracy (depending on the cut-off) and, on the other hand, to an increased demand on the number of simulated particles to achieve an acceptable error band as sign for a reliable result.

However, to overcome this methodological question, we have applied different approximations and simulation strategies to create trust in the results, specifically:

- the extraction method, which suffers from high computational demand due to the high number of isotopes in the calculation set
- the additive approach, which is computationally much more efficient, but the interaction between different chemical elements is neglected
- the cut-off of isotopes with low molar factions, which is unavoidable to create calculation results in acceptable time

The effect of all three approaches/approximations has been investigated and their combined evaluation led to an, in our view, statistically reliable result which is reflected in the overlap of the error bands for all investigated elements in the Monte-Carlo simulations with different setting creating trust in the applied method.

## Priority list and opportunities

### Optimization potential

The application of a continuous clean-up process opens completely new optimization opportunities since it allows a complete reshaping of the processes using innovative technologies and economic optimization processes. Using these opportunities efficiently opens the prospect of developing disruptive, cost optimized innovation into the nuclear fuel cycle, producing technologies which achieve a significant cost reduction due to the reduced challenge in the separation chemistry. The major points are:

- unlike in classical reprocessing there is no 99+% separation requirement to avoid losses into the waste stream
- there are less preparation processes (fuel storage, transport, reprocessing, fuel production) required to close the fuel cycle
- there is optimization potential between throughput and separation factor to allow cost saving approaches

This changes in the general scheme of the fuel cycle open an optimization potential which has finally to be studied in an integral simulation representing the reactor operation and the linked fuel cycle facility to be able to follow the streams of material and the consequences of the changed streams on the reactor behaviour and the salt composition in the reactor. However, a first indicative view can be taken of the following optimization parameters and their consequences on the reactor composition and operation:

- The number of steps/stages, each additional stage has a lower yield than the previous one–it is an asymptotic problem
- Throughput through the clean-up process–it influences the asymptotic amount of the elements in the reactor salt
- Combination of stages and throughput depending on the requested preparation–this is an economic optimization, cost of volume versus cost of components
- The acceptable concentration of an element to allow long term reactor operation–some elements can simply stay in the system and will accumulate during operation without causing harm

**Fig 7:**
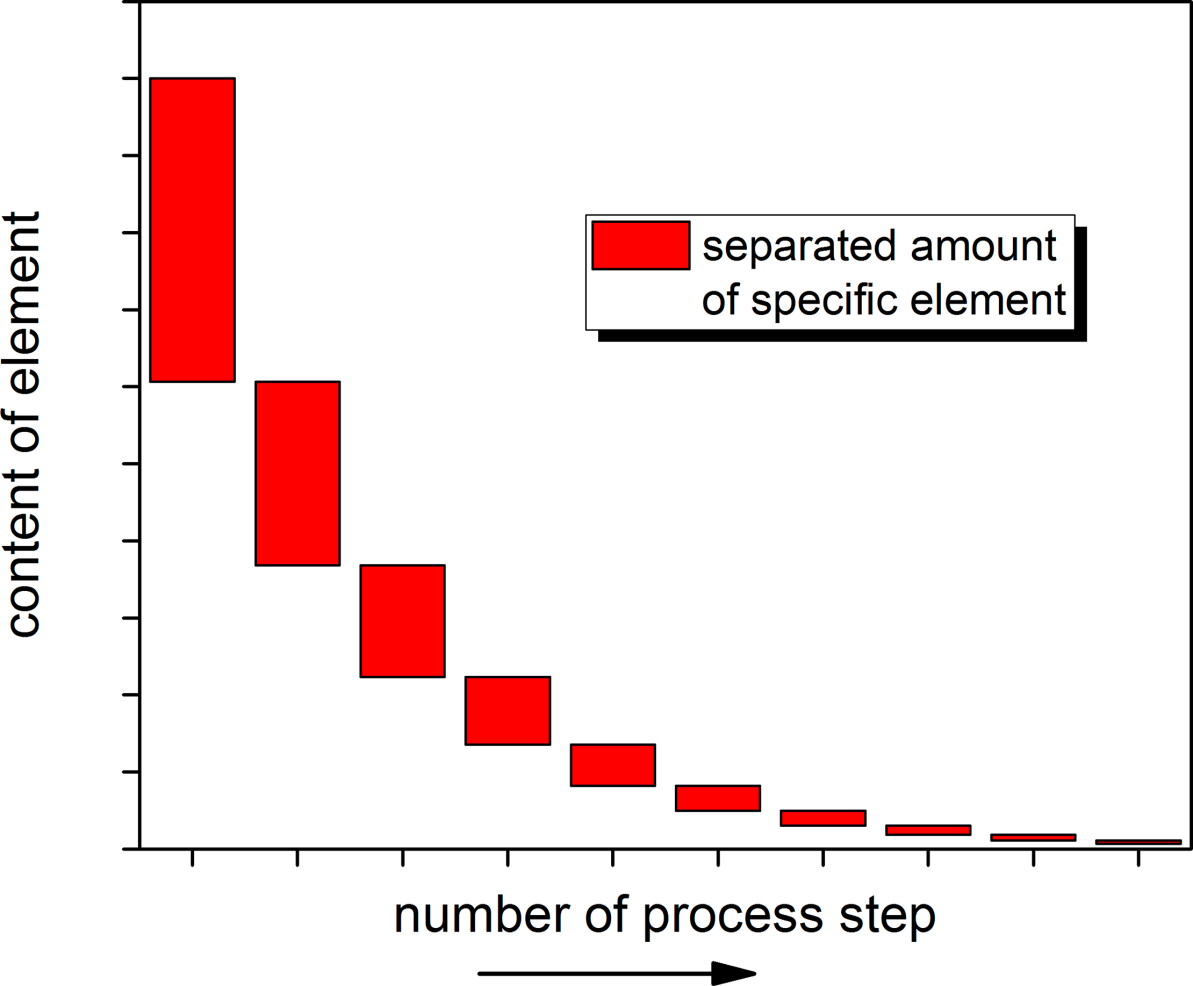
Qualitative view on the efficiency of separation stages.

A qualitative analysis of the efficiency of multiple separation stages shows that typically there is a decrease in the amount of an element separated per step. This is caused by the decreasing amount and proportion of the specific element which is present to be separated from the solution, see Fig 7. The figure has to be seen as illustrative example of the extent of the problem as the rate of decrease of the underlying exponential function, and hence the number of steps required for the required separation, is determined by the separation factor. However, this kind of asymptotic behaviour describes the challenge presented to the currently used reprocessing technology, which aims for very high separation factors up to 99.9%. In this scheme the last share is the lowest which will be separated and the effort to perform this last step is the highest due to the very low concentration of the target element. Thus any improvement of the overall separation factor becomes more costly the closer the result should be to 100%. The finally achieved separation factor per step is formed out of different variables of the process like:

- the theoretical achievable separation factor
- the contact area for the chemical reaction
- the time available for the reaction
- the initial concentration of the element at the begin of the reaction

**Fig 8:**
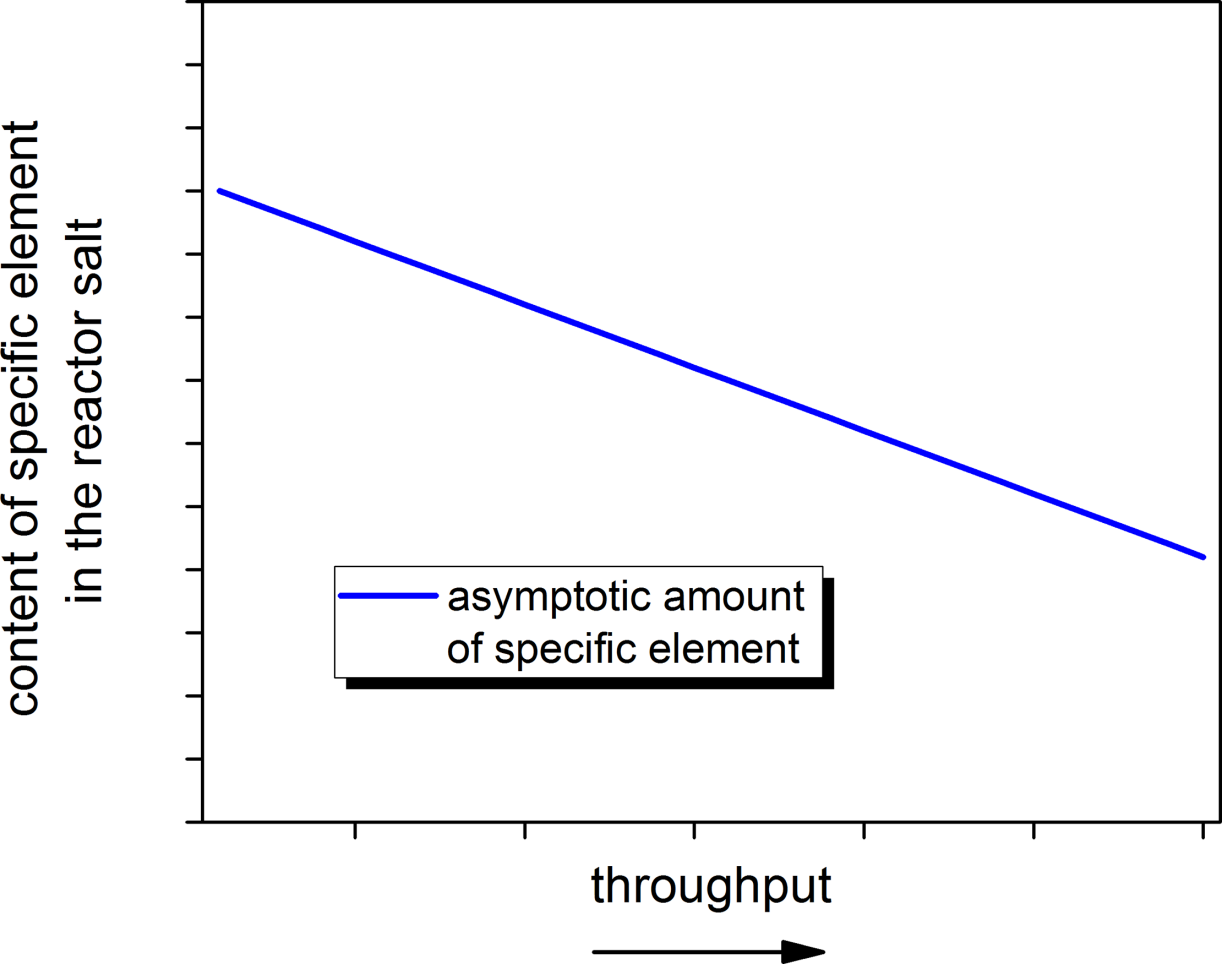
Qualitative view on the asymptotic level of content of an element in the fuel salt versus throughput in the clean-up system.

The described asymptotic behaviour explains why a breakthrough in fuel cycle technologies can result when the required separation factor can be significantly reduced. In this case only a few very efficient steps would be required, and ideally only one sufficiently efficient step. However, finally this will have to be compensated with a higher throughput since this is the second parameter which forms the asymptotic concentration of a specific element in the fuel salt in the reactor core. The asymptotic content of a specific element which is separated from the salt stream goes linear with the share of salt put through the salt clean-up system, see Fig 8. However, this is only true under the basic assumption that the separation factor is independent of the content of the specific element in the core. Taking into account Fig 7, it is obvious that the separation rate will decrease with lower average content, thus the linear behaviour will tend to an asymptotic behaviour for increasing throughput. The gradient of the reduction of the specific element depends on the production rate of the element due to the fission process and on the separation factor for the specific element in the clean-up system. The initial value is given by the amount of spent fuel inserted into the initial configuration and the content of the specific element in the spent fuel which is element characteristic but depends on the target burnup of the spent LWR fuel used in the molten salt reactor. However, to achieve a reasonable separation factor will still be a challenge due to the requirement for separation of trivalent lanthanide FPs from the trivalent TRU elements. This is particularly true for the Nd/Am pair, since Nd ranks high as an FP poison in the present study.

The final asymptotic amount of an element will depend on the throughput of salt per year through the salt clean-up, the amount of the element produced by the fission reaction in a specific time, and the separation factor achieved in the salt clean-up in one throughput. Summing up all these influence factors will lead to the request to have a low throughput and as high as possible content of the element in the fuel salt to make the clean-up system as efficient as possible. However, this exactly counteracts the request of the reactor physics problem for the elements which have a strong influence on the neutron economy–these elements should have as low as possible concentration in the fuel from point of view of reactor operation to allow sufficient breeding of fresh fissile material. Nevertheless, for all fission product elements which do not have a strong influence on the neutron economy, this strategy could be promising as long as the concentrations are well below the solubility limits.

The combination of throughput and separation efficiency is not only the optimization factor from physics and chemistry point of view, but also from economic point of view. The economy has to be optimized depending on the cost of installing several stages versus the cost per volume to be treated (batch size), the cost for the preparation of the salt for the processing stage and the cost of volume for the whole salt clean-up system. This will demands a multi-level economic optimization based on multi-disciplinary input during the development of the design of the requested separation steps for the salt clean-up system.

### New, demand driven approach

In a more generic view, the demand driven reprocessing technology and the related chemistry has to be designed on the basis of the reactor physics and chemistry instead of the waste characteristics and fissile material gain of the traditional reprocessing. The thinking has to change from:

- which elements have to be avoided to go into the final disposal?
- what is it worth to have this material once more available for fission?

to the new thinking

- which elements have the strongest influence on the neutron economy
- which of these elements is easiest to take out.
- which elements can be accepted to which extent to assure sustainable long term operation of the reactor

to identify the optimal steps for the optimization of the salt clean-up system.

### Reactor safety considerations

**Fig 9:**
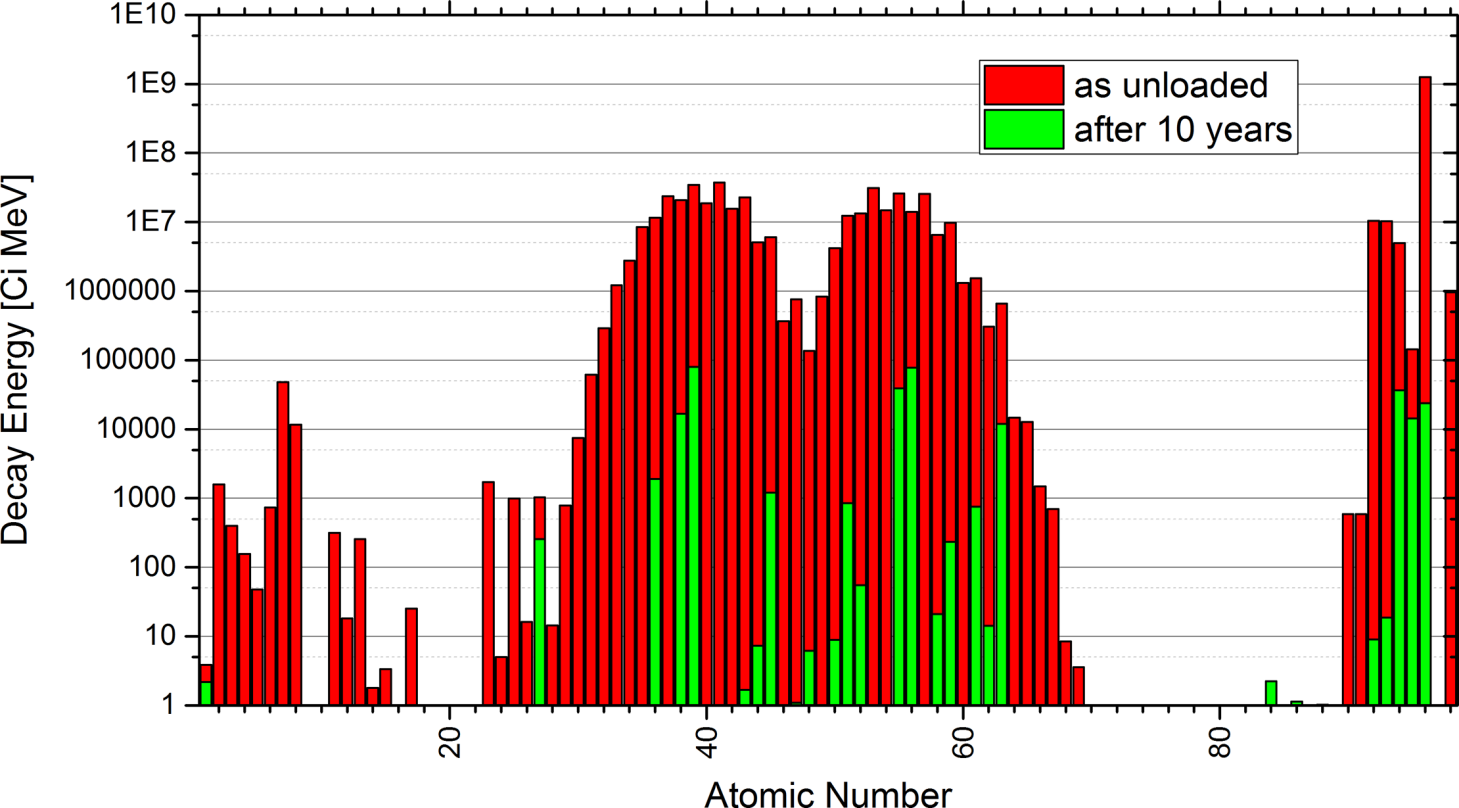
Comparison of the summed decay energy of SNF directly after unloading and after 10 years of storage based on calculation results of FISPIN.

Additional points can come from the reactor safety where the decay heat production and the radiological source term are important parameters. First indications for this parameters can be given by comparing the SNF composition directly after unloading from the reactor with the one after 10 years after unloading. Fig 9 indicates Curium as by far the major contributor to the summed decay heat directly after shutdown of a LWR. A fast reactor system has potential to change this since the Curium production in a fast reactor is typically lower than in a LWR. In general, the decay heat production directly after shutdown follows very close the fission yield curve, thus a significant change is not to be expected by the selective demand driven salt clean-up. For a more detailed investigation the development of the summed decay heat energy could give some hints to identify critical elements.

**Fig 10:**
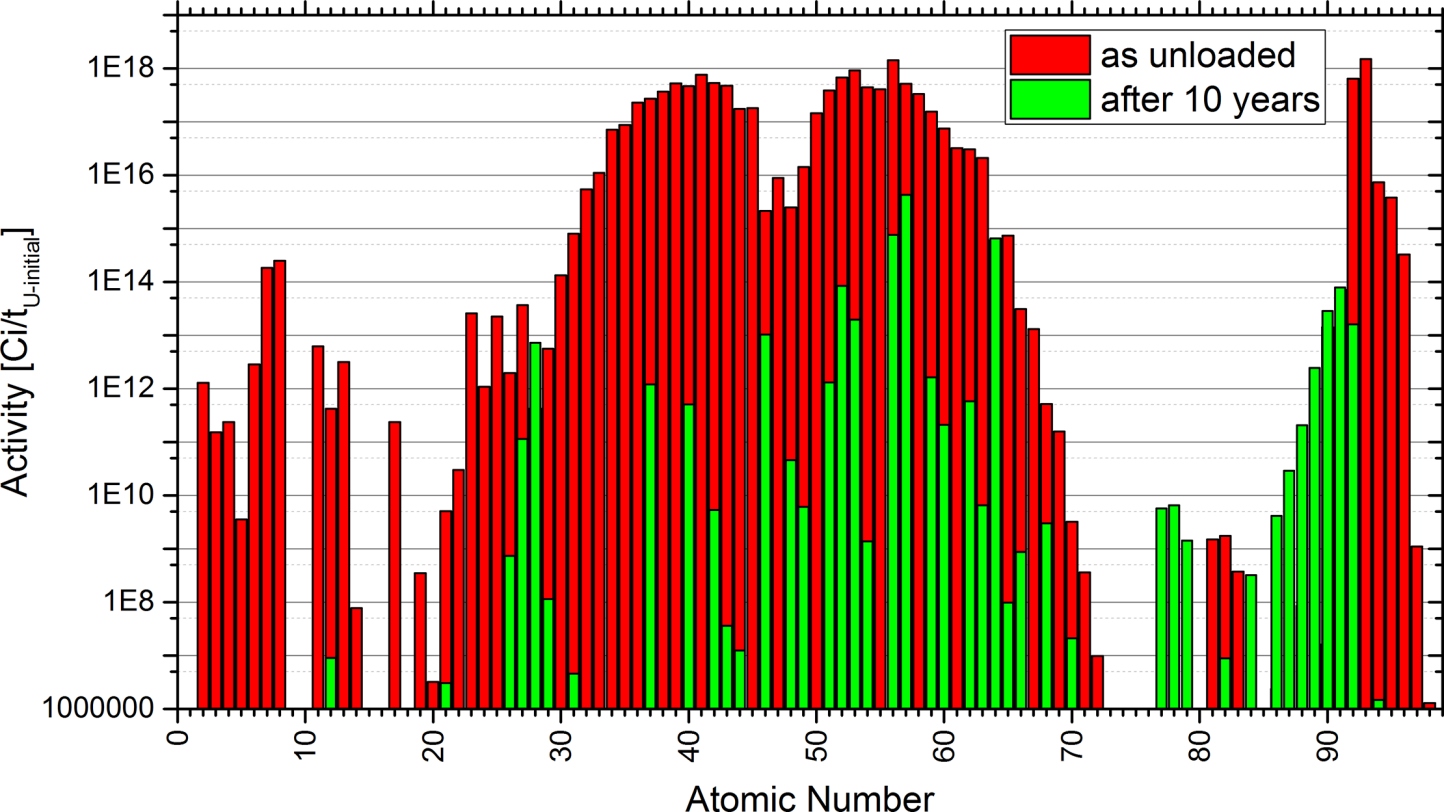
Comparison of the summed activity of SNF directly after unloading and after 10 years of storage based on calculation results of FISPIN.

The summed activity directly after shutdown (Fig 10) follows the fission yield distribution, too. A second peak is given by the actinides which have either been inserted or been bred in the reactor. Based on this distribution it is almost impossible to identify elements which could be attractive to be separated to reduce the activity level. A decision on separating elements to reduce the radiological source term would have been taken based on the time dependent reduction of the activity due to decay, on the radiological consequences of release of the elements in an accident leading to a release, and on possible release pathes. This will depend on possible accident scenarios as well on possible release scenarios which can only be identified when a detailed design of the reactor system including the salt clean-up is available.

However, some specific of molten salt reactors have to be kept in mind when discussing about the decay heat production and the radiological source term. A molten salt reactor won’t accumulate all fission products in the core. A significant part of the gaseous and volatile fission products will evaporate during operation and have to be captured in the off-gas system. Thus only a part of the sources will be in the core, another part will be accumulated in the off-gas system and has to be treated safely there.

## Conclusions and next steps

A short history of reprocessing technology focused on the PUREX technology based on aqueous processes has been given. This process is currently the leading reprocessing technology in the nuclear system of several major player in nuclear. The process scheme seems to be the ideal process for light water reactor fuel and the use of solid fuel–thus, it is the backbone of today’s industrial reprocessing. However, demand driven development of the nuclear system to fulfil the energy trilemma would favour a change to liquid fuelled reactors operating on spent nuclear fuel of light water reactors. This disruptive reactor technology opens the way/requires disruptive technology development in the separation technologies, too. Completely new possibilities are given due to the application of an integrated fuel cycle with a direct, online link to reactor operation. The possibilities for a new concept for innovative reprocessing/salt clean-up technology development are analysed, the boundary conditions are discussed, and the economic optimization parameters are worked out. The required reactor physical knowledge about the influence of different elements on the neutron economy of the reactor is created using an innovative study approach. Monte Carlo calculations based on the EVOL benchmark geometry are provided using a material configuration developed in the original proposal of the molten salt reactor operating on spent nuclear fuel from light water reactors. The variation of the fission product element contents is used to define an elementwise priority list for the salt clean-up. The priority list indicates Neodymium, Cesium and ideally Zirconium and probably Samarium as the most important elements to separate from the fuel salt. This will create a significant challenge given that the chemistry of Neodymium and Americium are nearly identical. This theoretical, reactor physical, study is followed by a qualitative discussion of the different, demand driven optimization strategies required to fulfil the conflicting interests consisting of sustainable long term reactor operation which requires on the one hand a neutron economy which allows sufficient breeding of fresh fissile material. On the other hand, efficient chemical processes for the salt clean-up which would be easier to implement with increasing concentration of the element to be separated. The related economic consequences of both parts are discussed. A new, innovative approach of balancing the throughput through the salt processing and the processing based on a low number of separation process steps is proposed to show the optimization potential of the salt clean-up of an integrated system. Finally, a short insight is given into points which can arise from the reactor safety where the decay heat production and the radiological source term are important parameters.

The next steps should include the development of chemical processes for the separation of Neodymium and Cesium, and preferably Zirconium and Samarium. This has to be supported by the development of an innovative process flow sheet for the salt clean-up which is able to link the different separation processes in the most convenient way. The developed process flow requires an optimization of the interplay between throughput, number of separation steps, and influence to the long term reactor behavior. It should include an economic study to identify the most cost effective combination of separation of specific elements, number of separation steps, throughput, and reactor operability. For the evaluation of the optimized system an integral, multi-disciplinary simulation tool for the integrated nuclear system has to be developed which is able to evaluate the reactor physical long term behavior in conjunction with the process modelling and the underlying chemistry.

An additional request for long term operation of the system is the identification of a low tech strategy for the separation of all fission product elements from the salt which do not have a strong influence on the neutron economy to avoid an accumulation which could lead to conflicts with the solubility limits.

## Supporting information

S1 File List of the isotopes in the spent nuclear fuel based on UOX fuel with burnup 50 GWd/tU after 10 years storage time. (DOCX)

S2 File Structural materials composition. (DOCX)

## Abbreviations

P&T: Partitioning and Transmutation
MSR: Molten Salt Reactor
MSFR: Molten Salt Fast Reactor
LWR: Light Water Reactor
AGR: Advanced Gas Cooled Reactor
SNF: Spent Nuclear Fuel
TRU: Transuranium–all elements with an atomic number higher than 92
HM: heavy metal–all elements with an atomic number higher than 90, typically the base to account for fissile materials in the reactor
GWd/t_HM_: Gigawattdays per ton of heavy metal–measure for the burnup of nuclear material in a reactor
GenIV: Reactor systems of the 4th generation as defined by the Generation IV International Forum
Δk_eff_: difference from the critical reactor status, typically measured in pcm (percent mille)
LiF: Lithium fluoride
FP: Fission products
PUREX: Plutonium Uranium Redox EXtraction
GANEX: Group actinide extraction
COEX: Co-extraction, trademark of AREVA
SANEX: Selective ActiNide EXtraction
DIAMEX: DIAMide Extraction
TALSPEAK: Trivalent Actinide Lanthanide Separation with Phosphorus-Reagent Extraction from Aqueous Komplexes
EVOL: European project for the development of a molten salt fast reactor
OpenMC: Reactor physics code based on the Monte Carlo method
JEFF-3.2: Nuclear data library
IAEA: International Atomic Energy Agency
