# Supplementary material for: Demand driven salt clean-up in a molten salt fast reactor – Defining a priority list
Source: PLoS One. 2018 Mar 1;13(3):e0192020. doi: 10.1371/journal.pone.0192020 (PMC5832222; doi:10.1371/journal.pone.0192020)
Supplement: S1 File — (DOCX) [file pone.0192020.s001.docx]

*List of the isotopes in the spent nuclear fuel based on UOX fuel with burnup 50 GWd/tU after 10 years storage time.*

| **Isotope** | **Mole** | **Activity** | **Atomic mass** |
| --- | --- | --- | --- |
| H1 | 4.68E-03 | 0.00E+00 | 4.68E-03 |
| H2 | 2.00E-03 | 0.00E+00 | 3.99E-03 |
| H3 | 1.32E-02 | 3.83E+02 | 3.97E-02 |
| HE3 | 1.23E-02 | 0.00E+00 | 3.70E-02 |
| HE4 | 4.30E-01 | 0.00E+00 | 1.72E+00 |
| LI6 | 6.48E-03 | 0.00E+00 | 3.89E-02 |
| LI7 | 4.04E-03 | 0.00E+00 | 2.83E-02 |
| BE7 | 1.45E-32 | 3.56E-26 | 1.02E-31 |
| BE9 | 2.44E-04 | 0.00E+00 | 2.19E-03 |
| BE10 | 1.40E-03 | 3.13E-04 | 1.40E-02 |
| B10 | 6.71E-07 | 0.00E+00 | 6.71E-06 |
| B11 | 1.65E-02 | 0.00E+00 | 1.81E-01 |
| C12 | 2.76E+00 | 0.00E+00 | 3.31E+01 |
| C13 | 1.62E+00 | 0.00E+00 | 2.10E+01 |
| C14 | 1.25E-02 | 7.82E-01 | 1.76E-01 |
| N14 | 6.91E-01 | 0.00E+00 | 9.67E+00 |
| N15 | 1.19E-02 | 0.00E+00 | 1.78E-01 |
| O16 | 8.38E+03 | 0.00E+00 | 1.34E+05 |
| O17 | 3.36E+00 | 0.00E+00 | 5.71E+01 |
| O18 | 1.68E+01 | 0.00E+00 | 3.02E+02 |
| F19 | 1.08E-01 | 0.00E+00 | 2.06E+00 |
| NE21 | 2.97E-04 | 0.00E+00 | 6.24E-03 |
| NE22 | 4.15E-06 | 0.00E+00 | 9.13E-05 |
| NA22 | 6.25E-10 | 8.59E-05 | 1.38E-08 |
| NA23 | 2.49E-01 | 0.00E+00 | 5.72E+00 |
| MG24 | 1.00E-01 | 0.00E+00 | 2.40E+00 |
| MG25 | 1.26E-02 | 0.00E+00 | 3.16E-01 |
| MG26 | 1.39E-02 | 0.00E+00 | 3.62E-01 |
| AL27 | 8.08E-01 | 0.00E+00 | 2.18E+01 |
| SI28 | 2.90E-01 | 0.00E+00 | 8.11E+00 |
| SI29 | 1.48E-02 | 0.00E+00 | 4.28E-01 |
| SI30 | 9.71E-03 | 0.00E+00 | 2.91E-01 |
| SI32 | 3.52E-10 | 7.33E-07 | 1.13E-08 |
| P31 | 1.73E-05 | 0.00E+00 | 5.36E-04 |
| P32 | 8.00E-14 | 7.33E-07 | 2.56E-12 |
| S32 | 2.93E-05 | 0.00E+00 | 9.38E-04 |
| S33 | 2.70E-08 | 0.00E+00 | 8.92E-07 |
| S34 | 6.85E-08 | 0.00E+00 | 2.33E-06 |
| S35 | 2.91E-18 | 4.35E-12 | 1.02E-16 |
| S36 | 2.09E-06 | 0.00E+00 | 7.52E-05 |
| CL35 | 3.53E-02 | 0.00E+00 | 1.24E+00 |
| CL36 | 3.56E-03 | 4.22E-03 | 1.28E-01 |
| CL37 | 1.24E-02 | 0.00E+00 | 4.58E-01 |
| AR36 | 9.38E-08 | 0.00E+00 | 3.38E-06 |
| AR38 | 4.83E-05 | 0.00E+00 | 1.84E-03 |
| AR39 | 6.97E-08 | 9.27E-05 | 2.72E-06 |
| AR40 | 1.63E-06 | 0.00E+00 | 6.50E-05 |
| K39 | 1.08E-08 | 0.00E+00 | 4.20E-07 |
| K40 | 3.45E-04 | 9.63E-08 | 1.38E-02 |
| K41 | 1.20E-05 | 0.00E+00 | 4.92E-04 |
| CA40 | 2.16E-01 | 0.00E+00 | 8.63E+00 |
| CA41 | 1.93E-04 | 6.69E-04 | 7.90E-03 |
| CA42 | 1.46E-03 | 0.00E+00 | 6.13E-02 |
| CA43 | 3.04E-04 | 0.00E+00 | 1.31E-02 |
| CA44 | 4.65E-03 | 0.00E+00 | 2.05E-01 |
| CA45 | 3.47E-13 | 2.78E-07 | 1.56E-11 |
| CA46 | 9.20E-06 | 0.00E+00 | 4.23E-04 |
| CA48 | 4.17E-04 | 0.00E+00 | 2.00E-02 |
| SC45 | 9.28E-06 | 0.00E+00 | 4.18E-04 |
| SC46 | 3.33E-21 | 5.19E-15 | 1.53E-19 |
| TI46 | 2.02E-07 | 0.00E+00 | 9.29E-06 |
| TI47 | 2.77E-07 | 0.00E+00 | 1.30E-05 |
| TI48 | 9.87E-11 | 0.00E+00 | 4.74E-09 |
| TI49 | 1.71E-06 | 0.00E+00 | 8.38E-05 |
| TI50 | 7.43E-07 | 0.00E+00 | 3.71E-05 |
| V50 | 7.22E-04 | 1.73E-15 | 3.61E-02 |
| V51 | 3.26E-01 | 0.00E+00 | 1.66E+01 |
| CR50 | 1.34E-02 | 0.00E+00 | 6.72E-01 |
| CR51 | 4.20E-45 | 1.83E-38 | 2.14E-43 |
| CR52 | 2.72E-01 | 0.00E+00 | 1.42E+01 |
| CR53 | 2.98E-02 | 0.00E+00 | 1.58E+00 |
| CR54 | 9.20E-03 | 0.00E+00 | 4.97E-01 |
| MN53 | 1.64E-07 | 1.58E-08 | 8.68E-06 |
| MN54 | 3.76E-08 | 1.57E-02 | 2.03E-06 |
| MN55 | 3.82E-02 | 0.00E+00 | 2.10E+00 |
| FE54 | 2.57E-01 | 0.00E+00 | 1.39E+01 |
| FE55 | 8.41E-05 | 1.11E+01 | 4.63E-03 |
| FE56 | 4.00E+00 | 0.00E+00 | 2.24E+02 |
| FE57 | 1.16E-01 | 0.00E+00 | 6.61E+00 |
| FE58 | 1.32E-02 | 0.00E+00 | 7.65E-01 |
| FE59 | 1.68E-30 | 4.86E-24 | 9.90E-29 |
| FE60 | 7.44E-07 | 3.54E-08 | 4.46E-05 |
| CO57 | 1.19E-11 | 5.71E-06 | 6.76E-10 |
| CO58 | 4.47E-21 | 8.25E-15 | 2.60E-19 |
| CO59 | 4.46E-02 | 0.00E+00 | 2.63E+00 |
| CO60 | 1.45E-03 | 9.81E+01 | 8.68E-02 |
| NI58 | 2.00E-01 | 0.00E+00 | 1.16E+01 |
| NI59 | 1.87E-03 | 8.92E-03 | 1.10E-01 |
| NI60 | 8.28E-02 | 0.00E+00 | 4.97E+00 |
| NI61 | 3.90E-03 | 0.00E+00 | 2.38E-01 |
| NI62 | 1.05E-02 | 0.00E+00 | 6.49E-01 |
| NI63 | 2.90E-04 | 1.04E+00 | 1.83E-02 |
| NI64 | 2.98E-03 | 0.00E+00 | 1.91E-01 |
| CU63 | 2.67E-02 | 0.00E+00 | 1.68E+00 |
| CU65 | 1.20E-02 | 0.00E+00 | 7.80E-01 |
| ZN64 | 8.35E-03 | 0.00E+00 | 5.35E-01 |
| ZN65 | 2.50E-10 | 1.33E-04 | 1.62E-08 |
| ZN66 | 4.81E-03 | 0.00E+00 | 3.17E-01 |
| ZN67 | 6.69E-04 | 0.00E+00 | 4.48E-02 |
| ZN68 | 3.20E-03 | 0.00E+00 | 2.18E-01 |
| ZN70 | 1.31E-04 | 0.00E+00 | 9.17E-03 |
| GA69 | 1.28E-05 | 0.00E+00 | 8.83E-04 |
| GA71 | 6.79E-05 | 0.00E+00 | 4.82E-03 |
| GE70 | 5.91E-09 | 0.00E+00 | 4.14E-07 |
| GE72 | 1.59E-04 | 0.00E+00 | 1.15E-02 |
| GE73 | 3.82E-04 | 0.00E+00 | 2.79E-02 |
| GE74 | 1.06E-03 | 0.00E+00 | 7.85E-02 |
| GE76 | 8.70E-03 | 0.00E+00 | 6.61E-01 |
| AS73 | 6.86E-29 | 1.12E-22 | 5.01E-27 |
| AS75 | 2.31E-03 | 0.00E+00 | 1.73E-01 |
| SE74 | 5.52E-10 | 0.00E+00 | 4.09E-08 |
| SE75 | 3.35E-24 | 3.65E-18 | 2.51E-22 |
| SE76 | 8.68E-05 | 0.00E+00 | 6.60E-03 |
| SE77 | 1.50E-02 | 0.00E+00 | 1.16E+00 |
| SE78 | 4.06E-02 | 0.00E+00 | 3.16E+00 |
| SE79 | 8.82E-02 | 4.85E-01 | 6.97E+00 |
| SE80 | 2.32E-01 | 0.00E+00 | 1.85E+01 |
| SE82 | 6.14E-01 | 1.58E-15 | 5.04E+01 |
| BR79 | 1.11E-05 | 0.00E+00 | 8.80E-04 |
| BR81 | 3.86E-01 | 0.00E+00 | 3.13E+01 |
| BR82 | 1.77E-23 | 1.58E-15 | 1.45E-21 |
| KR78 | 4.86E-14 | 0.00E+00 | 3.79E-12 |
| KR80 | 2.72E-06 | 0.00E+00 | 2.18E-04 |
| KR81 | 5.31E-07 | 9.04E-07 | 4.30E-05 |
| KR82 | 1.09E-02 | 0.00E+00 | 8.94E-01 |
| KR83 | 7.22E-01 | 0.00E+00 | 5.99E+01 |
| KR84 | 1.95E+00 | 0.00E+00 | 1.64E+02 |
| KR85 | 2.25E-01 | 7.51E+03 | 1.92E+01 |
| KR86 | 3.24E+00 | 0.00E+00 | 2.78E+02 |
| RB83 | 2.49E-22 | 3.77E-16 | 2.06E-20 |
| RB84 | 8.95E-42 | 3.57E-35 | 7.52E-40 |
| RB85 | 1.99E+00 | 0.00E+00 | 1.69E+02 |
| RB87 | 4.13E+00 | 3.08E-05 | 3.60E+02 |
| SR84 | 2.45E-08 | 0.00E+00 | 2.06E-06 |
| SR85 | 3.74E-27 | 7.53E-21 | 3.18E-25 |
| SR86 | 8.50E-03 | 0.00E+00 | 7.31E-01 |
| SR87 | 3.87E-05 | 0.00E+00 | 3.37E-03 |
| SR88 | 5.68E+00 | 0.00E+00 | 5.00E+02 |
| SR89 | 5.48E-23 | 1.42E-16 | 4.88E-21 |
| SR90 | 6.99E+00 | 8.58E+04 | 6.29E+02 |
| Y88 | 2.97E-15 | 3.64E-09 | 2.61E-13 |
| Y89 | 7.63E+00 | 0.00E+00 | 6.79E+02 |
| Y89M | 1.88E-32 | 1.32E-20 | 1.67E-30 |
| Y90 | 1.75E-03 | 8.58E+04 | 1.58E-01 |
| Y91 | 8.19E-20 | 1.83E-13 | 7.45E-18 |
| ZR90 | 2.33E+00 | 0.00E+00 | 2.10E+02 |
| ZR91 | 9.77E+00 | 0.00E+00 | 8.89E+02 |
| ZR92 | 1.04E+01 | 0.00E+00 | 9.60E+02 |
| ZR93 | 1.14E+01 | 2.67E+00 | 1.06E+03 |
| ZR94 | 1.23E+01 | 0.00E+00 | 1.16E+03 |
| ZR95 | 5.30E-18 | 1.08E-11 | 5.03E-16 |
| ZR96 | 1.25E+01 | 0.00E+00 | 1.20E+03 |
| NB91 | 3.52E-13 | 1.85E-10 | 3.20E-11 |
| NB92 | 1.02E-09 | 1.04E-11 | 9.40E-08 |
| NB93 | 1.34E-05 | 0.00E+00 | 1.25E-03 |
| NB93M | 4.79E-05 | 1.05E+00 | 4.46E-03 |
| NB94 | 1.40E-04 | 2.46E-03 | 1.31E-02 |
| NB95 | 6.45E-18 | 2.40E-11 | 6.13E-16 |
| NB95M | 3.32E-21 | 1.20E-13 | 3.15E-19 |
| MO92 | 7.19E-03 | 0.00E+00 | 6.62E-01 |
| MO93 | 1.35E-05 | 1.38E-03 | 1.25E-03 |
| MO94 | 4.48E-03 | 0.00E+00 | 4.22E-01 |
| MO95 | 1.16E+01 | 0.00E+00 | 1.10E+03 |
| MO96 | 1.13E+00 | 0.00E+00 | 1.09E+02 |
| MO97 | 1.22E+01 | 0.00E+00 | 1.19E+03 |
| MO98 | 1.29E+01 | 0.00E+00 | 1.26E+03 |
| MO100 | 1.41E+01 | 0.00E+00 | 1.41E+03 |
| TC97 | 2.12E-09 | 2.92E-10 | 2.06E-07 |
| TC97M | 6.64E-23 | 9.74E-17 | 6.44E-21 |
| TC98 | 1.94E-03 | 1.65E-04 | 1.90E-01 |
| TC99 | 1.16E+01 | 1.95E+01 | 1.15E+03 |
| RU98 | 3.56E-09 | 0.00E+00 | 3.49E-07 |
| RU99 | 8.37E-04 | 0.00E+00 | 8.29E-02 |
| RU100 | 1.91E+00 | 0.00E+00 | 1.91E+02 |
| RU101 | 1.14E+01 | 0.00E+00 | 1.15E+03 |
| RU102 | 1.20E+01 | 0.00E+00 | 1.22E+03 |
| RU103 | 5.81E-29 | 1.93E-22 | 5.99E-27 |
| RU104 | 8.16E+00 | 0.00E+00 | 8.49E+02 |
| RU106 | 2.06E-03 | 7.30E+02 | 2.18E-01 |
| RH101 | 1.78E-18 | 1.93E-13 | 1.80E-16 |
| RH102 | 5.60E-05 | 6.91E+00 | 5.72E-03 |
| RH102M | 5.99E-16 | 3.78E-10 | 6.11E-14 |
| RH103 | 6.18E+00 | 0.00E+00 | 6.36E+02 |
| RH103M | 5.75E-32 | 1.93E-22 | 5.92E-30 |
| RH106 | 1.93E-09 | 7.30E+02 | 2.04E-07 |
| PD102 | 7.64E-11 | 0.00E+00 | 7.79E-09 |
| PD104 | 3.70E+00 | 0.00E+00 | 3.85E+02 |
| PD105 | 5.67E+00 | 0.00E+00 | 5.96E+02 |
| PD106 | 5.07E+00 | 0.00E+00 | 5.38E+02 |
| PD107 | 2.96E+00 | 1.63E-01 | 3.17E+02 |
| PD108 | 2.03E+00 | 0.00E+00 | 2.19E+02 |
| PD110 | 6.04E-01 | 0.00E+00 | 6.64E+01 |
| AG107 | 8.04E-04 | 0.00E+00 | 8.61E-02 |
| AG108 | 1.53E-15 | 1.22E-04 | 1.66E-13 |
| AG108M | 1.60E-06 | 1.37E-03 | 1.73E-04 |
| AG109 | 7.02E-01 | 0.00E+00 | 7.65E+01 |
| AG109M | 7.80E-16 | 2.22E-04 | 8.51E-14 |
| AG110 | 1.11E-14 | 5.09E-03 | 1.22E-12 |
| AG110M | 7.33E-07 | 3.83E-01 | 8.06E-05 |
| CD106 | 3.52E-05 | 0.00E+00 | 3.73E-03 |
| CD108 | 2.67E-04 | 0.00E+00 | 2.89E-02 |
| CD109 | 2.50E-09 | 7.03E-04 | 2.72E-07 |
| CD110 | 7.92E-01 | 0.00E+00 | 8.71E+01 |
| CD111 | 3.14E-01 | 0.00E+00 | 3.49E+01 |
| CD112 | 1.53E-01 | 0.00E+00 | 1.72E+01 |
| CD113 | 1.15E-03 | 4.47E-14 | 1.30E-01 |
| CD113M | 1.28E-03 | 3.26E+01 | 1.45E-01 |
| CD114 | 1.67E-01 | 0.00E+00 | 1.90E+01 |
| CD115M | 4.30E-29 | 1.26E-22 | 4.94E-27 |
| CD116 | 6.39E-02 | 0.00E+00 | 7.41E+00 |
| IN113 | 9.41E-04 | 0.00E+00 | 1.06E-01 |
| IN114 | 2.03E-33 | 3.19E-22 | 2.32E-31 |
| IN114M | 1.26E-28 | 3.33E-22 | 1.44E-26 |
| IN115 | 2.03E-02 | 1.42E-11 | 2.33E+00 |
| IN115M | 1.98E-35 | 1.38E-26 | 2.28E-33 |
| SN112 | 1.80E-04 | 0.00E+00 | 2.02E-02 |
| SN113 | 1.76E-16 | 2.00E-10 | 1.99E-14 |
| SN114 | 1.39E-04 | 0.00E+00 | 1.58E-02 |
| SN115 | 3.04E-03 | 0.00E+00 | 3.50E-01 |
| SN116 | 4.42E-02 | 0.00E+00 | 5.12E+00 |
| SN117 | 5.95E-02 | 0.00E+00 | 6.96E+00 |
| SN118 | 6.23E-02 | 0.00E+00 | 7.35E+00 |
| SN119 | 6.23E-02 | 0.00E+00 | 7.41E+00 |
| SN119M | 1.24E-08 | 5.54E-03 | 1.48E-06 |
| SN120 | 6.41E-02 | 0.00E+00 | 7.69E+00 |
| SN121 | 2.35E-07 | 2.72E+01 | 2.84E-05 |
| SN121M | 4.90E-03 | 3.50E+01 | 5.93E-01 |
| SN122 | 8.84E-02 | 0.00E+00 | 1.08E+01 |
| SN123 | 8.43E-12 | 8.52E-06 | 1.04E-09 |
| SN124 | 1.57E-01 | 0.00E+00 | 1.94E+01 |
| SN126 | 3.37E-01 | 1.21E+00 | 4.25E+01 |
| SB121 | 5.79E-02 | 0.00E+00 | 7.00E+00 |
| SB123 | 9.77E-02 | 0.00E+00 | 1.20E+01 |
| SB124 | 7.31E-22 | 1.59E-15 | 9.07E-20 |
| SB125 | 1.22E-02 | 1.59E+03 | 1.52E+00 |
| SB126 | 1.60E-08 | 1.69E-01 | 2.02E-06 |
| SB126M | 1.22E-10 | 1.21E+00 | 1.54E-08 |
| TE121 | 4.16E-19 | 3.24E-12 | 5.03E-17 |
| TE121M | 3.89E-18 | 3.30E-12 | 4.71E-16 |
| TE122 | 4.56E-03 | 0.00E+00 | 5.56E-01 |
| TE123 | 6.02E-05 | 2.15E-12 | 7.40E-03 |
| TE123M | 7.26E-19 | 7.92E-13 | 8.93E-17 |
| TE124 | 8.91E-03 | 0.00E+00 | 1.10E+00 |
| TE125 | 2.03E-01 | 0.00E+00 | 2.54E+01 |
| TE125M | 1.73E-04 | 3.89E+02 | 2.16E-02 |
| TE126 | 6.97E-03 | 0.00E+00 | 8.79E-01 |
| TE127 | 4.38E-15 | 1.47E-06 | 5.56E-13 |
| TE127M | 1.25E-12 | 1.50E-06 | 1.59E-10 |
| TE128 | 1.11E+00 | 0.00E+00 | 1.42E+02 |
| TE129 | 2.58E-38 | 6.96E-29 | 3.33E-36 |
| TE129M | 2.75E-35 | 1.07E-28 | 3.55E-33 |
| TE130 | 4.32E+00 | 0.00E+00 | 5.62E+02 |
| I127 | 5.04E-01 | 0.00E+00 | 6.40E+01 |
| I129 | 2.05E+00 | 4.67E-02 | 2.65E+02 |
| XE124 | 1.36E-22 | 0.00E+00 | 1.68E-20 |
| XE126 | 2.93E-05 | 0.00E+00 | 3.69E-03 |
| XE127 | 3.23E-37 | 1.16E-30 | 4.11E-35 |
| XE128 | 4.82E-02 | 0.00E+00 | 6.16E+00 |
| XE129 | 3.69E-04 | 0.00E+00 | 4.76E-02 |
| XE130 | 1.12E-01 | 0.00E+00 | 1.45E+01 |
| XE131 | 3.63E+00 | 0.00E+00 | 4.76E+02 |
| XE132 | 1.35E+01 | 0.00E+00 | 1.78E+03 |
| XE134 | 1.68E+01 | 0.00E+00 | 2.25E+03 |
| XE136 | 2.45E+01 | 0.00E+00 | 3.33E+03 |
| CS133 | 1.22E+01 | 0.00E+00 | 1.63E+03 |
| CS134 | 5.28E-02 | 9.15E+03 | 7.07E+00 |
| CS135 | 4.60E+00 | 7.15E-01 | 6.21E+02 |
| CS137 | 1.05E+01 | 1.25E+05 | 1.44E+03 |
| BA132 | 4.57E-05 | 0.00E+00 | 6.04E-03 |
| BA133 | 1.82E-10 | 6.20E-06 | 2.42E-08 |
| BA134 | 2.12E+00 | 0.00E+00 | 2.84E+02 |
| BA135 | 4.03E-03 | 0.00E+00 | 5.44E-01 |
| BA136 | 3.28E-01 | 0.00E+00 | 4.46E+01 |
| BA137 | 3.28E+00 | 0.00E+00 | 4.50E+02 |
| BA137M | 1.60E-06 | 1.18E+05 | 2.20E-04 |
| BA138 | 1.42E+01 | 0.00E+00 | 1.96E+03 |
| LA137 | 1.11E-06 | 6.61E-06 | 1.52E-04 |
| LA138 | 2.18E-03 | 7.42E-09 | 3.01E-01 |
| LA139 | 1.31E+01 | 0.00E+00 | 1.82E+03 |
| CE138 | 1.74E-09 | 0.00E+00 | 2.40E-07 |
| CE139 | 6.64E-16 | 6.30E-10 | 9.23E-14 |
| CE140 | 1.32E+01 | 0.00E+00 | 1.84E+03 |
| CE141 | 6.12E-35 | 2.46E-28 | 8.63E-33 |
| CE142 | 1.20E+01 | 8.56E-11 | 1.70E+03 |
| CE144 | 4.11E-04 | 1.88E+02 | 5.91E-02 |
| PR141 | 1.19E+01 | 0.00E+00 | 1.68E+03 |
| PR144 | 1.73E-08 | 1.88E+02 | 2.49E-06 |
| PR144M | 1.04E-10 | 2.82E+00 | 1.49E-08 |
| ND142 | 2.54E-01 | 0.00E+00 | 3.61E+01 |
| ND143 | 8.08E+00 | 0.00E+00 | 1.16E+03 |
| ND144 | 1.40E+01 | 2.38E-09 | 2.01E+03 |
| ND145 | 6.83E+00 | 0.00E+00 | 9.90E+02 |
| ND146 | 7.37E+00 | 0.00E+00 | 1.08E+03 |
| ND148 | 3.81E+00 | 0.00E+00 | 5.64E+02 |
| ND150 | 1.78E+00 | 0.00E+00 | 2.66E+02 |
| PM145 | 1.22E-09 | 2.47E-05 | 1.77E-07 |
| PM146 | 1.28E-04 | 8.29E+00 | 1.87E-02 |
| PM147 | 8.80E-02 | 1.20E+04 | 1.29E+01 |
| PM148 | 1.92E-31 | 4.68E-24 | 2.85E-29 |
| PM148M | 3.20E-29 | 1.01E-22 | 4.73E-27 |
| SM146 | 4.03E-04 | 1.40E-06 | 5.88E-02 |
| SM147 | 1.78E+00 | 6.02E-06 | 2.62E+02 |
| SM148 | 1.75E+00 | 8.96E-11 | 2.59E+02 |
| SM149 | 3.23E-02 | 5.77E-12 | 4.81E+00 |
| SM150 | 2.82E+00 | 0.00E+00 | 4.23E+02 |
| SM151 | 1.39E-01 | 5.59E+02 | 2.09E+01 |
| SM152 | 1.32E+00 | 0.00E+00 | 2.01E+02 |
| SM154 | 3.68E-01 | 0.00E+00 | 5.67E+01 |
| EU149 | 1.38E-22 | 1.94E-16 | 2.06E-20 |
| EU150 | 4.23E-08 | 4.42E-04 | 6.34E-06 |
| EU151 | 1.14E-02 | 0.00E+00 | 1.73E+00 |
| EU152 | 1.63E-04 | 4.38E+00 | 2.48E-02 |
| EU153 | 1.23E+00 | 0.00E+00 | 1.88E+02 |
| EU154 | 1.86E-01 | 7.75E+03 | 2.87E+01 |
| EU155 | 2.26E-02 | 1.63E+03 | 3.51E+00 |
| GD151 | 6.15E-17 | 6.47E-11 | 9.28E-15 |
| GD152 | 9.88E-04 | 3.27E-12 | 1.50E-01 |
| GD153 | 2.42E-09 | 1.31E-03 | 3.70E-07 |
| GD154 | 2.66E-01 | 0.00E+00 | 4.10E+01 |
| GD155 | 6.98E-02 | 0.00E+00 | 1.08E+01 |
| GD156 | 8.08E-01 | 0.00E+00 | 1.26E+02 |
| GD157 | 1.33E-03 | 0.00E+00 | 2.09E-01 |
| GD158 | 2.07E-01 | 0.00E+00 | 3.27E+01 |
| GD160 | 2.13E-02 | 0.00E+00 | 3.40E+00 |
| TB157 | 4.35E-09 | 1.58E-05 | 6.83E-07 |
| TB158 | 5.72E-06 | 1.13E-02 | 9.04E-04 |
| TB159 | 3.38E-02 | 0.00E+00 | 5.38E+00 |
| TB160 | 7.99E-19 | 1.44E-12 | 1.28E-16 |
| DY159 | 7.52E-19 | 6.80E-13 | 1.20E-16 |
| DY160 | 4.26E-03 | 0.00E+00 | 6.81E-01 |
| DY161 | 8.35E-03 | 0.00E+00 | 1.34E+00 |
| DY162 | 7.55E-03 | 0.00E+00 | 1.22E+00 |
| DY163 | 7.52E-03 | 0.00E+00 | 1.23E+00 |
| DY164 | 2.69E-03 | 0.00E+00 | 4.42E-01 |
| HO163 | 1.16E-09 | 9.08E-08 | 1.89E-07 |
| HO165 | 4.14E-03 | 0.00E+00 | 6.84E-01 |
| HO166M | 6.32E-05 | 1.88E-02 | 1.05E-02 |
| ER166 | 1.36E-03 | 0.00E+00 | 2.26E-01 |
| ER167 | 9.95E-05 | 0.00E+00 | 1.66E-02 |
| TM168 | 7.61E-21 | 1.07E-14 | 1.28E-18 |
| TM169 | 5.71E-05 | 0.00E+00 | 9.65E-03 |
| TM170 | 1.45E-16 | 1.47E-10 | 2.47E-14 |
| TM171 | 2.10E-07 | 3.90E-02 | 3.59E-05 |
| YB168 | 2.83E-12 | 0.00E+00 | 4.76E-10 |
| YB170 | 2.11E-07 | 0.00E+00 | 3.58E-05 |
| YB171 | 1.12E-05 | 0.00E+00 | 1.91E-03 |
| YB172 | 5.06E-06 | 0.00E+00 | 8.71E-04 |
| YB173 | 2.82E-06 | 0.00E+00 | 4.87E-04 |
| YB174 | 1.87E-06 | 0.00E+00 | 3.26E-04 |
| YB176 | 3.98E-07 | 0.00E+00 | 7.00E-05 |
| LU175 | 1.48E-06 | 0.00E+00 | 2.59E-04 |
| LU177 | 2.45E-20 | 4.78E-13 | 4.34E-18 |
| LU177M | 2.78E-18 | 2.25E-12 | 4.92E-16 |
| HF177 | 1.05E-07 | 0.00E+00 | 1.86E-05 |
| HF178 | 2.76E-08 | 0.00E+00 | 4.91E-06 |
| HF179 | 7.20E-09 | 0.00E+00 | 1.29E-06 |
| HF180 | 1.85E-09 | 0.00E+00 | 3.32E-07 |
| HF181 | 4.91E-37 | 1.51E-30 | 8.89E-35 |
| HF182 | 1.25E-10 | 4.95E-12 | 2.27E-08 |
| TA181 | 4.90E-10 | 0.00E+00 | 8.86E-08 |
| TA182 | 4.36E-18 | 4.95E-12 | 7.93E-16 |
| W 182 | 1.03E-16 | 0.00E+00 | 1.88E-14 |
| W 183 | 2.74E-11 | 0.00E+00 | 5.01E-09 |
| HG201 | 7.38E-12 | 0.00E+00 | 1.48E-09 |
| HG202 | 1.89E-13 | 0.00E+00 | 3.81E-11 |
| HG204 | 3.34E-09 | 0.00E+00 | 6.82E-07 |
| HG206 | 5.78E-26 | 1.33E-15 | 1.19E-23 |
| TL204 | 4.03E-11 | 3.82E-06 | 8.23E-09 |
| TL205 | 3.39E-10 | 0.00E+00 | 6.95E-08 |
| TL206 | 4.46E-23 | 2.00E-12 | 9.19E-21 |
| TL207 | 2.12E-15 | 8.36E-05 | 4.39E-13 |
| TL208 | 1.03E-12 | 6.35E-02 | 2.15E-10 |
| TL209 | 3.38E-18 | 2.89E-07 | 7.07E-16 |
| TL210 | 6.72E-22 | 9.71E-11 | 1.41E-19 |
| PB204 | 3.06E-04 | 0.00E+00 | 6.25E-02 |
| PB205 | 2.89E-06 | 6.79E-08 | 5.92E-04 |
| PB206 | 5.29E-03 | 0.00E+00 | 1.09E+00 |
| PB207 | 4.85E-03 | 0.00E+00 | 1.00E+00 |
| PB208 | 1.15E-02 | 0.00E+00 | 2.39E+00 |
| PB209 | 1.39E-14 | 1.34E-05 | 2.90E-12 |
| PB210 | 4.38E-12 | 7.02E-08 | 9.20E-10 |
| PB211 | 1.61E-14 | 8.41E-05 | 3.41E-12 |
| PB212 | 6.00E-10 | 1.77E-01 | 1.27E-07 |
| PB214 | 6.62E-17 | 4.64E-07 | 1.42E-14 |
| BI209 | 2.07E-07 | 0.00E+00 | 4.33E-05 |
| BI210 | 2.70E-15 | 7.03E-08 | 5.66E-13 |
| BI210M | 1.60E-11 | 1.90E-12 | 3.35E-09 |
| BI211 | 9.69E-16 | 8.40E-05 | 2.04E-13 |
| BI212 | 5.69E-11 | 1.77E-01 | 1.21E-08 |
| BI213 | 3.25E-15 | 1.34E-05 | 6.91E-13 |
| BI214 | 4.90E-17 | 4.63E-07 | 1.05E-14 |
| BI215 | 2.66E-21 | 6.75E-11 | 5.71E-19 |
| PO209 | 1.42E-20 | 4.99E-17 | 2.98E-18 |
| PO210 | 6.78E-14 | 6.40E-08 | 1.42E-11 |
| PO211 | 1.05E-20 | 2.29E-07 | 2.21E-18 |
| PO212 | 3.01E-21 | 1.13E-01 | 6.38E-19 |
| PO213 | 4.87E-24 | 1.31E-05 | 1.04E-21 |
| PO214 | 6.76E-24 | 4.62E-07 | 1.45E-21 |
| PO215 | 1.33E-20 | 8.42E-05 | 2.86E-18 |
| PO216 | 2.27E-15 | 1.77E-01 | 4.91E-13 |
| PO218 | 7.53E-18 | 4.64E-07 | 1.64E-15 |
| AT215 | 2.98E-27 | 3.36E-10 | 6.41E-25 |
| AT217 | 3.84E-20 | 1.34E-05 | 8.33E-18 |
| AT218 | 1.32E-23 | 9.29E-11 | 2.87E-21 |
| AT219 | 3.33E-22 | 6.96E-11 | 7.30E-20 |
| RN217 | 7.69E-26 | 1.61E-09 | 1.67E-23 |
| RN218 | 2.88E-28 | 9.27E-14 | 6.27E-26 |
| RN219 | 2.96E-17 | 8.43E-05 | 6.48E-15 |
| RN220 | 8.71E-13 | 1.77E-01 | 1.92E-10 |
| RN222 | 1.36E-14 | 4.64E-07 | 3.02E-12 |
| FR221 | 3.50E-16 | 1.34E-05 | 7.73E-14 |
| FR223 | 1.35E-16 | 1.16E-06 | 3.00E-14 |
| RA223 | 7.38E-12 | 8.43E-05 | 1.65E-09 |
| RA224 | 4.90E-09 | 1.77E-01 | 1.10E-06 |
| RA225 | 1.52E-12 | 1.34E-05 | 3.42E-10 |
| RA226 | 2.08E-09 | 4.64E-07 | 4.70E-07 |
| RA228 | 2.44E-11 | 1.51E-06 | 5.55E-09 |
| AC225 | 1.03E-12 | 1.34E-05 | 2.31E-10 |
| AC227 | 5.12E-09 | 8.41E-05 | 1.16E-06 |
| AC228 | 2.97E-15 | 1.51E-06 | 6.78E-13 |
| TH227 | 1.19E-11 | 8.31E-05 | 2.70E-09 |
| TH228 | 9.41E-07 | 1.76E-01 | 2.15E-04 |
| TH229 | 2.75E-07 | 1.34E-05 | 6.31E-05 |
| TH230 | 2.65E-05 | 1.25E-04 | 6.08E-03 |
| TH231 | 1.65E-10 | 2.03E-02 | 3.82E-08 |
| TH232 | 7.57E-02 | 1.93E-06 | 1.76E+01 |
| TH234 | 5.71E-08 | 3.09E-01 | 1.34E-05 |
| PA231 | 2.69E-05 | 2.94E-04 | 6.22E-03 |
| PA232 | 2.00E-16 | 1.99E-08 | 4.64E-14 |
| PA233 | 1.08E-07 | 5.23E-01 | 2.52E-05 |
| PA234 | 8.60E-13 | 4.02E-04 | 2.01E-10 |
| PA234M | 1.93E-12 | 3.09E-01 | 4.50E-10 |
| U232 | 3.49E-05 | 1.79E-01 | 8.09E-03 |
| U233 | 3.80E-03 | 8.54E-03 | 8.86E-01 |
| U234 | 5.46E-01 | 7.95E-01 | 1.28E+02 |
| U235 | 4.00E+01 | 2.03E-02 | 9.39E+03 |
| U235M | 5.42E-08 | 3.92E+02 | 1.27E-05 |
| U236 | 2.50E+01 | 3.81E-01 | 5.89E+03 |
| U237 | 1.44E-07 | 2.78E+00 | 3.41E-05 |
| U238 | 3.87E+03 | 3.09E-01 | 9.20E+05 |
| U240 | 5.23E-15 | 1.16E-06 | 1.26E-12 |
| NP236 | 5.29E-06 | 1.24E-05 | 1.25E-03 |
| NP237 | 3.13E+00 | 5.23E-01 | 7.41E+02 |
| NP238 | 6.51E-10 | 4.02E-02 | 1.55E-07 |
| NP239 | 6.46E-07 | 3.58E+01 | 1.54E-04 |
| NP240 | 4.02E-16 | 1.16E-06 | 9.65E-14 |
| PU236 | 6.38E-07 | 7.86E-02 | 1.51E-04 |
| PU237 | 1.13E-30 | 3.27E-24 | 2.69E-28 |
| PU238 | 1.36E+00 | 5.53E+03 | 3.23E+02 |
| PU239 | 2.64E+01 | 3.92E+02 | 6.31E+03 |
| PU240 | 1.08E+01 | 5.90E+02 | 2.60E+03 |
| PU241 | 4.58E+00 | 1.14E+05 | 1.10E+03 |
| PU242 | 3.21E+00 | 3.07E+00 | 7.77E+02 |
| PU243 | 7.96E-16 | 5.03E-07 | 1.93E-13 |
| PU244 | 2.61E-04 | 1.16E-06 | 6.36E-02 |
| PU246 | 5.45E-19 | 6.56E-12 | 1.34E-16 |
| AM241 | 3.05E+00 | 2.52E+03 | 7.35E+02 |
| AM242 | 4.54E-08 | 8.88E+00 | 1.10E-05 |
| AM242M | 3.52E-03 | 8.92E+00 | 8.52E-01 |
| AM243 | 7.37E-01 | 3.58E+01 | 1.79E+02 |
| AM245 | 3.25E-20 | 4.96E-11 | 7.95E-18 |
| AM246M | 8.72E-22 | 6.56E-12 | 2.15E-19 |
| CM241 | 1.10E-41 | 4.37E-35 | 2.64E-39 |
| CM242 | 9.22E-06 | 7.39E+00 | 2.23E-03 |
| CM243 | 2.24E-03 | 2.67E+01 | 5.44E-01 |
| CM244 | 2.01E-01 | 3.97E+03 | 4.91E+01 |
| CM245 | 1.93E-02 | 8.10E-01 | 4.72E+00 |
| CM246 | 2.13E-03 | 1.61E-01 | 5.24E-01 |
| CM247 | 2.25E-05 | 5.03E-07 | 5.56E-03 |
| CM248 | 1.58E-06 | 1.66E-06 | 3.91E-04 |
| CM250 | 4.89E-13 | 2.19E-11 | 1.22E-10 |
| BK249 | 8.39E-12 | 3.42E-06 | 2.09E-09 |
| CF249 | 2.59E-08 | 2.64E-05 | 6.46E-06 |
| CF250 | 4.40E-09 | 1.20E-04 | 1.10E-06 |
| CF251 | 2.69E-09 | 1.07E-06 | 6.74E-07 |
| CF252 | 1.18E-10 | 1.59E-05 | 2.97E-08 |
