# Supplementary material for: Demand driven salt clean-up in a molten salt fast reactor – Defining a priority list
Source: PLoS One. 2018 Mar 1;13(3):e0192020. doi: 10.1371/journal.pone.0192020 (PMC5832222; doi:10.1371/journal.pone.0192020)
Supplement: S2 File — (DOCX) [file pone.0192020.s002.docx]

*Structural materials composition*

The walls of the blanket region and reflectors of the model are made of the Ni-based alloy. The composition of the alloy was taken from [14] and is presented in Table 2.

| Element | Ni | W | Cr | Mo | Fe | Ti | C | Mn | Si | Al | B | P | S |
| --- | --- | --- | --- | --- | --- | --- | --- | --- | --- | --- | --- | --- | --- |
| Percentage | 79.432 | 9.976 | 8.014 | 0.736 | 0.632 | 0.295 | 0.294 | 0.257 | 0.252 | 0.052 | 0.033 | 0.023 | 0.004 |

Table 4. Composition (at%) of the Ni-based alloy used in the study

The density of the alloy was equal to 10 g/cm^3^.

The composition of the natural boron was: 19.8% of ^10^B and 80.2% of ^11^B. The density of the boron carbide (B_4_C) used as protector in this study was equal to 2.52016 g/cm^3^.
